# Clinical Identification of Dysregulated Circulating microRNAs and Their Implication in Drug Response in Triple Negative Breast Cancer (TNBC) by Target Gene Network and Meta-Analysis

**DOI:** 10.3390/genes12040549

**Published:** 2021-04-09

**Authors:** Amal Qattan, Taher Al-Tweigeri, Wafa Alkhayal, Kausar Suleman, Asma Tulbah, Suad Amer

**Affiliations:** 1 Breast Cancer Research, Department of Molecular Oncology, King Faisal Specialist Hospital and Research Centre, Riyadh 11211, Saudi Arabia; suad@kfshrc.edu.sa; 2 Department of Biochemistry and Molecular Medicine, School of Medicine and Health Sciences (SMHS), George Washington University, Washington, DC 20073, USA; 3 Department of Medical Oncology, Oncology Centre, King Faisal Specialist Hospital and Research Centre, Riyadh 11211, Saudi Arabia; ttwegieri@kfshrc.edu.sa (T.A.-T.); ksuleman@kfshrc.edu.sa (K.S.); 4 Department of Surgery, King Faisal Specialist Hospital and Research Centre, Riyadh 11211, Saudi Arabia; wkhayal@kfshrc.edu.sa; 5 Department of Pathology, King Faisal Specialist Hospital and Research Centre, Riyadh 11211, Saudi Arabia; tulbah@kfshrc.edu.sa

**Keywords:** circulating miRNA, triple-negative breast cancer, chemoresistance, theranostic markers

## Abstract

Resistance to therapy is a persistent problem that leads to mortality in breast cancer, particularly triple-negative breast cancer (TNBC). MiRNAs have become a focus of investigation as tissue-specific regulators of gene networks related to drug resistance. Circulating miRNAs are readily accessible non-invasive potential biomarkers for TNBC diagnosis, prognosis, and drug-response. Our aim was to use systems biology, meta-analysis, and network approaches to delineate the drug resistance pathways and clinical outcomes associated with circulating miRNAs in TNBC patients. MiRNA expression analysis was used to investigate differentially regulated circulating miRNAs in TNBC patients, and integrated pathway regulation, gene ontology, and pharmacogenomic network analyses were used to identify target genes, miRNAs, and drug interaction networks. Herein, we identified significant differentially expressed circulating miRNAs in TNBC patients (miR-19a/b-3p, miR-25-3p, miR-22-3p, miR-210-3p, miR-93-5p, and miR-199a-3p) that regulate several molecular pathways (PAM (PI3K/Akt/mTOR), HIF-1, TNF, FoxO, Wnt, and JAK/STAT, PD-1/PD-L1 pathways and EGFR tyrosine kinase inhibitor resistance (TKIs)) involved in drug resistance. Through meta-analysis, we demonstrated an association of upregulated miR-93, miR-210, miR-19a, and miR-19b with poor overall survival outcomes in TNBC patients. These results identify miRNA-regulated mechanisms of drug resistance and potential targets for combination with chemotherapy to overcome drug resistance in TNBC. We demonstrate that integrated analysis of multi-dimensional data can unravel mechanisms of drug-resistance related to circulating miRNAs, particularly in TNBC. These circulating miRNAs may be useful as markers of drug response and resistance in the guidance of personalized medicine for TNBC.

## 1. Introduction

Resistance to therapy is a persistent problem that leads to mortality in cancer patients. Specifically, breast cancer typically responds well to platinum based first-line therapy, but recurs with a resistant phenotype in the majority of cases. While multiple mechanisms can play into cancer drug resistance, such as decreased uptake of drugs, increased export of drugs, and alterations in cell cycle and DNA damage regulation, miRNAs have come to the forefront as tissue-specific regulators of entire gene networks related to drug resistance [1,2]. Dysregulation of complex genetic and functional networks by miRNAs are attractive mechanisms for therapeutic targeting to restore normal tissue function. MiRNAs are dysregulated in the tumor microenvironment and released into the bloodstream [3,4,5,6]. These circulating miRNAs are stable owing to structural resistance to RNases, making them amenable to applications as non-invasive markers of disease and drug response [7]. The accumulation of evidence for dysregulation of microRNAs (miRNAs) in triple negative breast cancer (TNBC) makes these markers particularly amenable to addressing the problem of drug resistance in this clinically challenging breast cancer subtype. Such markers will be especially actionable if identifiable in circulation.

Breast cancer is categorized according to hormone and growth factor receptor expression: progesterone receptor (PR), estrogen receptor (ER), and the epidermal growth factor receptor, HER2. These markers guide therapy and inform prognosis. While HER2-positive tumors can be effectively treated with HER2-targeted therapy, they tend to be aggressive [8]. Hormone receptor positive tumors are associated with better survival [9]. Breast tumors that are negative for all three markers are classified as TNBC, which accounts for 10–15% of breast cancer [10]. Chemotherapy is standard for TNBC, but this tumor type is plagued by significant drug-resistance [11,12,13,14]. Standard therapy regimens depend on subtype, with neoadjuvant and/or adjuvant platinum-based combinations being standard for TNBC [11]. Risk of mortality is 2.6-fold higher in hormone receptor-negative patients compared to other breast cancer subtypes [9]. Survival signaling, drug efflux, hypoxia induced angiogenesis, stem phenotypes, cell cycle regulation, DNA damage response, immune regulation, and epithelial-mesenchymal transition (EMT) are among the factors contributing to drug resistance in TNBC [6,7,8]. These factors represent pathways and functions that are regulated by miRNAs and that may be exploited to overcome drug resistance. 

Circulating miRNAs in blood, plasma, and serum are readily accessible non-invasive biomarkers for the diagnosis and prognosis of cancer, including TNBC, and its follow-up. Holubekova et al. recently found miR-99a, miR-130a, miR-484, and miR-1260a to be up-regulated in the plasma of breast cancer patients and suggested the deregulation of miRNAs involved in TGF-beta pathways and Hippo signaling pathways; although this study involved mostly luminal breast cancer subtypes and only two TNBC cases [15]. A study by Sahlberg et al. identified a four-miRNA signature (miR-107, miR-18b, miR-652, and miR-103) in circulation predicting relapse and overall survival in TNBC [16]. More recently, Kahraman et al. identified circulating miRNAs diagnostic of TNBC versus healthy controls (miR-126-5p) and response to platinum-based neoadjuvant therapy (miR-34a) [17]. Another recent study by Ritter et al. showed an association between serum miR-17, miR-19b, and miR-30 with response to neoadjuvant therapy in TNBC. However, a clear picture of the drug resistance-related circulating miRNAs that are relevant to the spectrum of drugs used for TNBC has yet to be revealed.

Preclinically, several miRNAs have been shown to act synergistically with chemotherapy drugs for the inhibition or killing of tumor cells [18,19,20]. The therapeutic use of miRNAs has begun to gain traction in clinical trials [13,14,15]. Combination therapy with miRNAs may present an opportunity to mitigate drug resistance in TNBC. Important molecular pathways related to tumorigenesis and drug resistance in TNBC include receptor tyrosine kinases (RTKs), PAM (PI3K/Akt/mTOR), Nf-kB/PI3K/STAT3/IL6, PTEN/PI3K/Akt, MAPK signaling, JACK/Stat, FoxO, Wnt, Hippo, and VEGF. These pathways have been exploited for the development of targeted therapies [6,7,8]. However, of these mechanisms, those that may be identifiable using circulating miRNA markers and targeted by the exploitation of miRNA therapies is largely unknown. Unique markers and targets for drug resistance related to circulating miRNAs in TNBC remain to be clarified. 

The purpose of this study was to identify circulating miRNAs that are differentially regulated in TNBC versus normal breast and luminal breast cancer subtypes and investigate the clinical relationship between specific circulating miRNAs, drug resistance pathways, and clinical outcomes. The overarching goal was to identify circulating markers that are specific to the triple negative subtype and may be useful in the guidance of personalized therapy for TNBC, based on association with drug response. Herein, we identify significant differentially expressed circulating miRNAs in TNBC patients that regulate several of the above-listed molecular pathways and analyze associated gene-miRNA networks that are related to resistance to drugs used to treat TNBC. Pooled survival meta-analysis using data from several biorepositories demonstrated significant associations of several of the miRNAs identified herein with overall survival in breast cancer. The research and clinical implications of this work include the delineation of miRNA-regulated drug resistance pathways and identification of markers for personalized therapy, potential targets, and approaches for overcoming drug resistance in TNBC by using or targeting miRNAs therapeutically in combination with chemotherapy. Herein, we demonstrate the potential for integrated analysis of multi-dimensional data to reveal drug resistance related circulating miRNAs in cancers, particularly in TNBC. This provides a compelling rationale for the further investigation of specific miRNA targets for combination therapy. 

## 2. Materials and Methods

### 2.1. Patient Specimen Accrual, Sample Collection, and Study Design 

The study followed a standard protocol approved by the Clinical Research Committee in the Office Research Affairs (ORA) and was performed in accordance with the Helsinki Declaration. Blood samples of 34 normal healthy individuals and 93 breast cancer patients, including 36 TNBC, 16 Luminal A, and 41 Luminal B cancers, were collected at the hospital by venesection in EDTA tubes (BD Vacutainer, Plymouth, UK) and kept at 4 °C. Control blood samples were collected from healthy individuals. All breast cancer samples were collected before surgery and therapy. Plasma samples were centrifuged, filtered, aliquoted, barcoded, and cryopreserved at −80 °C. All samples were de-identified and entered into a restricted access database. Electronic medical records (ICIS and power chart) and charts were used for collecting clinical information of patients under (RAC approval: 2160029). Aliquots of plasma from the samples were thawed only once, mixed, and centrifuged before processing.

### 2.2. Isolation of Total RNA and miRNAs Analysis

Circulating miRNAs were extracted from 200 µL aliquots of plasma using a miRNeasy Serum/Plasma Kit (Qiagen, Hilden, Germany), as proposed by Qattan et al. [21]. The quality and integrity of RNA samples were evaluated using a RNA 6000 Nano LabChip on an Agilent 2100 Bioanalyzer (USA). RNA eluted in RNase-free water was stored at −80 °C until use. RNA samples (250 ng) were reverse transcribed using miScript HiSpec buffer (Qiagen, Hilden, Germany). MiRNA profiling was performed on all 127 samples in the study using a MIHS-109Z miScript miRNA Array Human panel (Qiagen, Hilden, Germany) which comprises 84 mature miRNAs that are relevant to breast cancer. The plate was loaded into a RT-PCR system using the amplification mode (95 **°**C for 15 min, 40 cycles at 94 **°**C for 15 s, 55 °C for 30 s, and 70 °C for 30 s) and at the end, a melting curve program was followed. All samples were run in duplicate and were checked for positive PCR control (PPC) and reverse transcription controls (RTC), to check the efficiency of reverse transcription and cellular contamination. The kit was spiked with two internal miR-39 controls for normalization. The quality and efficiencies of each miRNA probe were determined using calibration curves. Correlation coefficients (R2) and PCR efficiency was calculated from the slopes, which were between 0.97–0.99. For each miRNA, the total number of values that equaled zero, or that were greater than 35, across samples in the groups were counted. The miRNAs with these values in one-third of the samples or more in a given group were not used for further analysis. Thus, after employing a series of quality control measures and normalization, we obtained 54 miRNAs that were used for downstream analysis, as proposed by Qattan et al. [21]. These miRNAs and their corresponding log2 (fold-change) and BH adjusted *p*-values are provided for all the subgroups analyzed in this study in Supplementary Table S1.

### 2.3. Data Analysis and Bioinformatics

Significant differentially expressed miRNAs in the circulation of patients with different breast cancer subtypes compared to healthy normal patients were obtained using an unpaired t-test and multiple testing using the Benjamini–Hochberg (BH) method. The relative fold difference between any two groups was measured by 2^(−ΔΔCt)^, as proposed by Livak et al. [22]. The miRNAs with adjusted *p*-value < 0.05 and 1.5 fold-change, either positive or negative, were considered to be of sufficient magnitude to include in further analysis. For visualization purposes, fold-change was represented in log2 scale (Volcano plots). BH adjusted *p*-values were computed, and if both fold change and *p*-value (<0.05) met the specific criteria, they were considered significantly altered. All statistical analyses were performed using R, version R-4.0.3. The ComplexHeatmap, EnhancedVolcano, tidyverse, circlize, and ggplot2 packages from R were used for data visualization [23,24,25,26,27]. As the dCT values of miRNAs provided an inverse association to their actual expression level in samples, negative sign was introduced to the Z-normalized miRNA profile and visualized as a heatmap in this study, as proposed by Gu et al. [23]. Clustering and visualization of miRNA profiles as heatmaps with Euclidean distance based measurements was performed using the complex heatmap package of R. The log_2_ fold change of each miRNA was plotted against their corresponding –log_10_ (BH adjusted pvalue) for any two groups of normal, TNBC, or luminal breast tumors to get a volcano plot using the Enhanced Volcano and ggplot2 libraries. The diagnostic value of the miRNAs of interest was investigated by performing receiver operating characteristic (ROC) curves using the ROCR package. The area under the ROC curves (AUC) was used to evaluate the potential of selected miRNAs to discriminate the TNBC from normal healthy subjects. The miRNA expression quantification data of The Cancer Genome Atlas (TCGA) breast cancers was downloaded from the National Cancer Institute, Genomic Data Commons (NCI-GDC) data portal on December 2020 (https://portal.gdc.cancer.gov (accessed on 17 February 2021); Grossman et al., 2016) [28]. The miRNA data of the Cancer Genome Atlas Breast Invasive Carcinoma TCGA–BRCA cohort was log2 transformed, scaled, and then used for further downstream analysis.

Power analysis was conducted for continuous variables, i.e., mean circulating miRNA levels based on an expected variance and effect size established by preliminary studies. Using an alpha of 0.05, it was determined that a sample size of 76, including 35 TNBC samples, 35 luminal, and 32 healthy controls, would achieve an 80% power to detect the smallest anticipated relative effect size with a beta probability of 0.2.

### 2.4. Gene-Set Enrichment (GSEA) and Drug Prediction Analysis

Target gene prediction for the miRNAs of interest and subsequent characterization of enriched gene ontology and pathway terms were performed using miRWalk3 and its in-built GSEA option. All possible miRNA binding sites within the complete sequence (3′-UTR, 5′-UTR, and CDS) of a gene were considered and analyzed. Pharmaco-miR, which contains data from the Pharmacogenomics Knowledge base (PharmGKB), Drug Bank, and various miRNA target prediction tools (TargetScan, miRTarBase, miRanda), was used to investigate the interactions between miRNA, coding transcripts, and drugs [29,30,31].

## 3. Results

### 3.1. Patient Characteristics

A total of 127 individuals were analyzed in this study, including 34 healthy subjects and 93 breast cancer patients; 36 of those classified as having TNBC. Invasive ductal carcinoma was the predominant histology, found in 84% of breast cancer patients. Subjects displayed grade 3 (62.37%) or grade 2 (30.11%) disease. Overall, 38.71% of breast cancer patients were TNBC (Table 1). 2.19 × 10^−10^.

### 3.2. Circulating microRNA Expression Profile in Human TNBC Samples 

**Figure 1:**
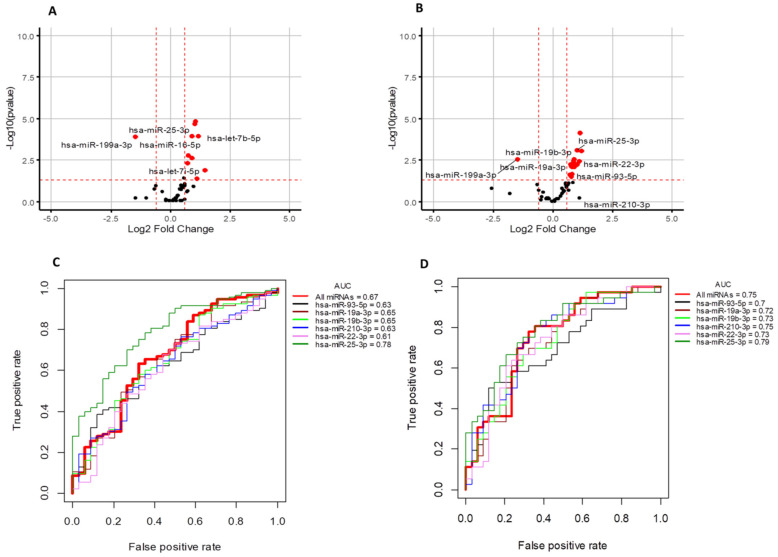
(**A**,**B**) Volcano plots showing the log2 fold difference between samples form healthy controls (n = 34) and breast cancer patients (n = 93) on the x-axis versus a measure of statistical significance (–log10 adjusted *p*-value) on the y-axis. Each point in the plot refers to a specific miRNA. The miRNAs that satisfied the criteria with adjusted *p*-value (BH) <0.05 are marked and highlighted as red marks in the figure. The miRNAs depicted in a black color did not have a significant adj-pvalue (BH). (**A**) Significantly regulated miRNAs in all breast tumors compared to control samples. (**B**) Significantly regulated miRNAs in TNBC compared to control samples. (**C**,**D**) AUC/ROC analysis (area under the curve/receiver operating characteristic curve) of significant differentially regulated miRNAs. (**C**) All breast cancers vs. control, (**D**) TNBC vs. control samples. AUC/ROC curves corresponding to individual miRNAs are plotted with different colors. The thick red in each plot represents the cumulative average expression of all miRNAs. See Table S2, Figure S3, and Supplementary Figure S4 for complementary data.

**Figure 2:**
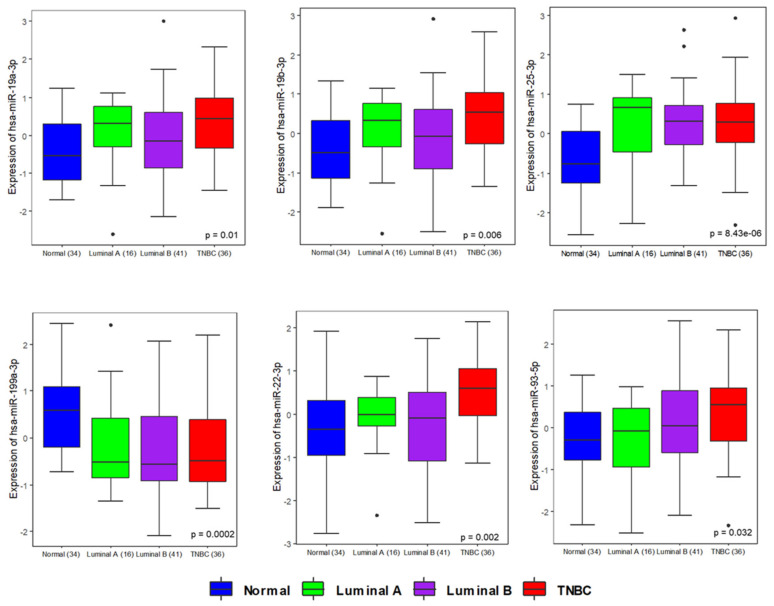
Expression pattern of miRNAs in different subtypes of breast cancer and normal healthy individuals. Individual boxplots show the expression pattern of miRNA smiR-19a-3p, miR-19b-3p, miR-199a-3p, miR-25-3p, miR-22-3p, and miR-93-5p across normal, luminal A, luminal B, and TNBC samples profiled in this study. Significant ANOVA-based *p*-values are indicated.

To identify miRNAs that were differentially present in the plasma of TNBC patients compared to healthy controls, and how the profile of deregulated circulating miRNAs differed by breast cancer type, we performed a qRT-PCR panel to analyze the relative abundance of 84 breast cancer-related miRNAs in the plasma of cancer cases (n = 93) and healthy controls (n = 34). While these miRNAs are implicated in breast cancer, this panel provides a platform for the discovery of subsets of breast cancer related miRNAs that may be specific to TNBC (n = 36) and that regulate, or are associated with, drug resistance. MiRNA abundance in plasma was compared to the miRNA sequencing (RNASeq) data that is publicly accessible from TCGA (downloaded from NCI Genomic data Commons (GDC) portal) to analyze trends of expression in both plasma and tissue samples. The expression of primary transcripts (precursors) was extracted from TCGA, and Euclidean distance based clustering was performed. In all, 1180 samples, including solid tissue samples of breast cancer (n = 1076) and disease-free individuals (n = 104), were used for this analysis. A heatmap with Euclidean distance-based clustering of miRNAs and TCGA samples is shown in Supplementary Figure S1. One-way Euclidean distance-based clustering of significant differentially expressed miRNAs across our breast cancer samples is shown in Supplementary Figure S2. Then, we analyzed the expression trends of a given miRNA across tissue and plasma samples. The tissue level expression of miRNA precursors was available as reads per kilobase of transcript per million (RPKM). The RPKM of the precursors in tissue and the expression value for miRNAs in the current study were log2 transformed and auto-scaled, to ensure the datasets were comparable. Auto scaled value = (x − μ)/δ. The tissue RPKM and the plasma cell-corrected Ct values were normalized to the mean (μ) and standard deviation (δ) for each of the datasets. Since a miRNA precursor can give rise to an active form from each arm, we compared both the 3′ and 5′ active forms matched to the same precursor. The differentially expressed active miRNA forms were mapped to precursor miRNAs from TCGA. Then we analyzed the expression trends of a given miRNA across tissue and plasma samples. Expression trends for some miRNAs were similar in both tissue and plasma. ( Figures S1–S3). Volcano plots highlighting the significance and magnitude of expression differences in miRNAs in breast cancer and TNBC versus healthy controls are presented in Figure 1A,B. These analyses revealed miR-19a/b-3p, miR-25–3p, miR-22-3p, miR-93-5p, and miR-210-3p as significantly upregulated plasma miRNAs and miR-199a-3p as a significantly downregulated plasma miRNA that were specific to the TNBC subtype; see Figure 1 and Supplementary Figure S4. Differentially expressed circulating miRNAs in breast cancer subtypes as detected by this analysis are presented in Table S2. qRT–PCR data demonstrating the differential expression of these significant miRNAs in TNBC, luminal A breast cancer, and luminal B breast cancer compared to normal controls are illustrated by whisker plots in Figure 2, and an AUC/ROC (area under the curve/receiver operating characteristic curve) analysis is presented in Figure 1C,D and Figure S5. Upon this validation of the deregulation of these miRNAs in the circulation of patients with TNBC, we considered these to be potential specific TNBC markers that may be related to disease characteristics, and as warranting further investigation. 

### 3.3. Analysis of the Function of the Targets of Significant Circulating miRNAs in Triple-Negative Breast Cancer

The KEGG (Kyoto Encyclopedia of Genes and Genomes) pathways, enriched according to differentially expressed mRNAs in TNBC patients within the complete sequence and possible miRNA binding sites (3′-UTR, 5′-UTR and CDS), were analyzed. Selection of signaling pathways with adjusted *p*-values (BH) ≤0.05 identified 60 unique 3′-UTR, 8 unique 5′-UTR, and 28 unique CDS-targeted pathways. KEGG pathway analysis revealed several molecular pathways that were enriched based on significant differentially expressed circulating miRNAs in TNBC, as illustrated in Figures S6 and S7. Specific significantly enriched pathways that are related to drug resistance [12] included PI3K/Akt/mTOR pathway, autophagy, EGFR tyrosine kinase inhibitor resistance (TKIs), PD-1/PD-L1 pathway, cellular senescence, P53, HIFa, TNF, FoxO, VEGF, Wnt, JACK/STAT, cell cycle, platinum drug, MAPK, AMPK, and Hippo; see Figure S7 and Table 2. Multiple cancer-relevant pathways were regulated by significantly differentially expressed miRNAs in TNBC, including PI3K-Akt, cell-cycle, TKIs, PD-1/PD-L1 pathway, mTOR, and JAK-STAT signaling (Supplementary Figures S11–S17). The hierarchical clustering of miRNAs and associated KEGG pathways demonstrated significant pathways associated with differentially regulated circulating miRNAs in TNBC (Supplementary Figure S18). Several drug resistance pathways were broadly regulated by multiple significant miRNAs found in TNBC patients, including PI3K-Akt signaling, cell cycle, EGFR tyrosine kinase inhibitor resistance (TKIs), autophagy, PD-1/PD-L1 pathway in cancer, MAPK and AMPK signaling, Wnt signaling, JAK-STAT signaling, and TGF-beta signaling.

To delineate the cellular components, biological processes, and molecular functions associated with differentially expressed circulating miRNAs in TNBC, gene ontology analysis was performed. Table 2 represents KEGG enriched pathways associated with significant miRNAs in triple negative breast cancer (TNBC) among genes with miRNA binding sites within complete sequences (3′-UTR, 5′-UTR, and CDS). For comparison, significant gene ontology with adjusted *p*-values (BH) ≤0.05, enriched in 3′-UTR, 5′-UTR and CDS specific entries for TNBC and for luminal breast cancer are presented in Figures S8 and S9.

Three widely used classifications were used for Gene Ontology (GO) ontologies, including cellular component, biological process, and molecular function categories. A threshold of an adjusted *p*-value (BH) ≤0.05 was used to indicate statistical significance. We identified 1108 unique genes with 3′-UTR targets, 161 with 5′-UTR targets, and 923 with CDS target sequences. Enriched GO terms (BP–MF–CC) in TNBC patients within the complete sequence and possible miRNA binding sites (3′-UTR, 5′-UTR, and CDS) corresponded to the significant terms of the adjusted *p*-value (BH) ≤0.05. Enriched GO–CC terms comprised 150 unique 3′-UTR terms, including nuclear chromatin, transcriptional repressor complex, and midbody; 7 unique 5′-UTR terms, including receptor complex, focal adhesion, and mitochondrial matrix; and 133 unique CDS terms, including nuclear membrane, integral components of endoplasmic reticulum membrane, mitotic spindle, nuclear body, cell cortex, and polysome. Regulated genes were found to be involved in a number of GO–BP (biological processes), including 488 unique GO–BP (3′-UTR), 16 unique GO–BP (5′-UTR), and 370 unique GO„BP (CDS), which are closely associated with tumorigenesis and metastasis. Gene ontology enrichment analyses revealed that the target genes of these miRNAs were mainly associated with the top GO–BP terms of cell cycle, cell division, negative regulation of apoptotic processes, cellular response to DNA damage stimulus, cellular response to hypoxia, negative regulation of G0–G1 transition, G1–S transition of mitotic cell cycle, positive regulation of the Wnt signaling pathway, cellular response to glucose starvation, cytokine mediated signaling pathway, positive regulation of mitotic cell cycle phase transition, and regulation of transcription from RNA polymerase II promoter in response to hypoxia (Supplementary Figure S10 and Table 3). Among the enriched GO–MF terms, there were 144 unique 3′-UTR, 11 unique 5′-UTR, and 125 unique CDS. Overall, common and highly enriched functions in both the KEGG pathway and GO analyses included cell cycle regulation, survival function, DNA repair, and proliferative signaling. 

### 3.4. Significant TNBC-Specific miRNAs Regulating Genes Involved in Cancer Drug Resistance 

**Figure 3:**
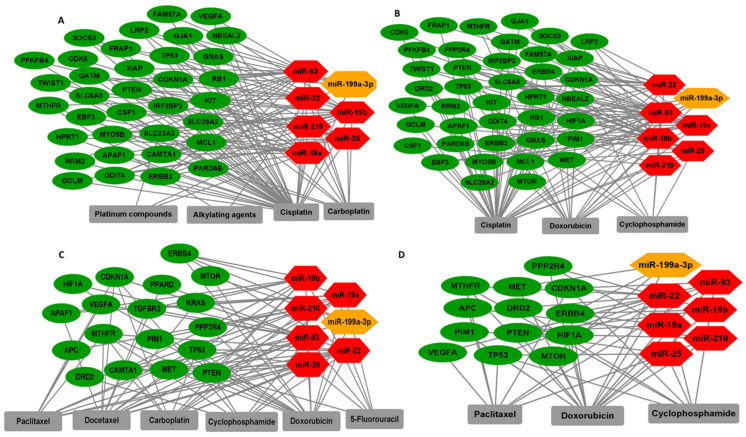
Interactions among different drug combinations for TNBC therapy with significantly upregulated miRNAs (red), downregulated miRNAs (orange), and their target genes (green) for: (**A**) platinum compounds, alkylating agents, cisplatin, and carboplatin; (**B**) cisplatin, doxorubicin, and cyclophosphamide; (**C**) paclitaxel, docetaxel, carboplatin, cyclophosphamide, doxorubicin, and 5-flourouracil (5-FU); (**D**) doxorubicin, paclitaxel, and cyclophosphamide.

PharmGKB and drug bank with Pharmaco-miR was used to create networks between significant miRNAs and target genes involved in cancer drug resistance (Table 4). Networks related to select chemotherapy regimens including platinum, carboplatin, cisplatin, paclitaxel, doxorubicin, cyclophosphamide, and 5-flourouracil (5-FU), which are shown in Figure 3. We identified genes regulated by significant differentially expressed miRNAs in TNBC that affect both chemotherapies and targeted therapies, for example, TP53 and PTEN are regulated by miR-25 and related to docetaxel and doxorubicin resistance. TP53 is also regulated by miR-22 and is related to carboplatin resistance. VEGFA and CDKN1A are regulated by miR-93 and related to paclitaxel resistance. RUNX3, which is regulated by miR-19a/b, is related to fluorouracil resistance. Overexpression of RUNX3 caused resistance of epithelial ovarian cancer cells to carboplatin [32]. RUNX3 may also be related to chemoresistance in breast cancer. The function of RUNX3 in breast cancer remains unknown. Non-coding RNAs that target RUNX3 in breast cancer have been previously identified, suggesting that further investigation of the role of RUNX3 in chemoresistance and its regulation in breast cancer is justified.

### 3.5. Association of Significant Circulating miRNAs with the Survival of Breast Cancer Patients

**Figure 4:**
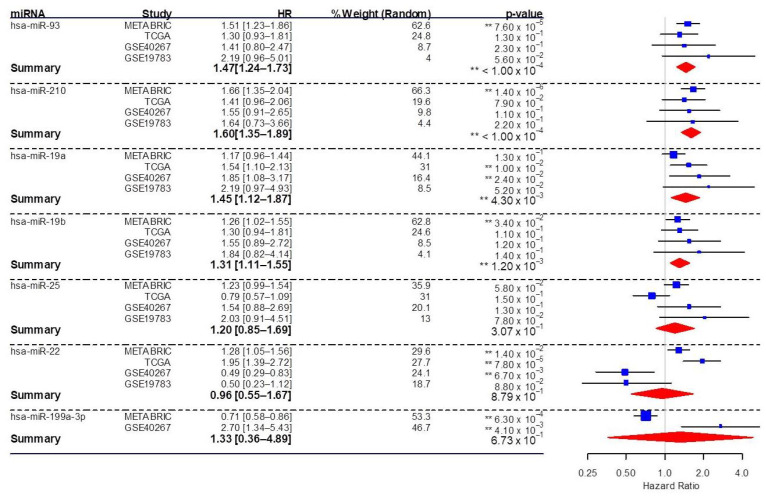
Forest plot showing the association of significant miRNAs in our study with overall survival from various breast cancer studies, including METABRIC, TCGA, GSE40267, and GSE19783. Hazard ratios (HR) and confidence intervals are presented. The relative weight of each study and pooled HR estimates were calculated using the random-effects model. Blue squares represent hazard ratios of individual miRNAs in different breast cancer studies (METABRIC, TCGA, GSE40267, and GSE19783). Red diamonds indicate pooled effect estimates of miRNAs using the random-effects model and a 95% confidence interval. A hazard ratio (HR) >1 indicates that as the miRNA presence in breast cancer increases, risk increases, and thus survival decreases, whereas a risk ratio <1 indicates a reduced risk and increased survival. HR, hazard ratio; TCGA, The Cancer Genome Atlas; METABRIC, Molecular Taxonomy of Breast Cancer International Consortium; miRNA, microRNA. **—*p*-value < 0.05.

We addressed the clinical significance of the significant circulating miRNAs identified in this study as potential theranostic markers. Meta-analysis of survival data was conducted using Kaplan–Meier plotter (KM Plotter) to investigate the impact of miRNA expression on overall survival in breast cancer patients. In all, 2502 breast cancer samples from various cohorts including METABRIC (n = 1262), TCGA (n = 1062), GSE40267 (n = 85), and GSE19783 (n = 93) were analyzed using KM Plotter. Meta-analysis using the random effect model was conducted to get the combined estimate of hazard ratios (HR). The increase in overall pooled effect (HR with 95% confidence interval) of the significant miRNAs indicated an increased risk of death in breast cancer patients (HR >1) associated with upregulation of miR-93, miR-210, miR-19a, and miR-19b (Figure 4). After the removal of non-TNBC samples, 203 TNBC samples from METABRIC and 97 from TCGA were analyzed. Even with this smaller sample size, miR-93 remained significant (*p* = 0.0468), with an HR of 1.56 (CI: 1.01–2.41). 

## 4. Discussion

Circulating miRNAs have been shown to have potential as biomarkers and therapeutic targets in both HER2-positive and triple-negative breast cancers [33]. Herein, we identified several circulating miRNAs that are differentially regulated in TNBC (miR-19a-3p, miR-19b-3p, miR-25-3p, miR-22-3p, miR-93-5p, miR-210-3p (upregulated) and miR-199a-3p (downregulated)) and regulate chemoresistance related gene networks. A meta-analysis revealed that overexpression of miR-93, miR-210, miR-19a, and miR-19b correlates with poor overall survival outcomes in TNBC patients. There is evidence of the involvement of several of these miRNAs mechanisms in cancer drug resistance. Regulation of cell cycle and proliferative signaling may be an important mechanism of miRNA on drug resistance. For example, miR-19a has been shown to regulate anti-tumor immunity [34,35,36]. MiR-19a and miR-19b can also activate NF-kB and repress PTEN, to exert an oncogenic effect in TNBC [21,22]. MiR-210 was up regulated in TNBC compared to luminal breast cancer. High hsa-miR-210 expression has been associated with poor prognosis for TNBC [37].

Our analysis of drug–miRNA–gene target networks identified specific chemotherapy regimens for which response may be associated with circulating miRNAs in the plasma of TNBC patients, and which we have distinguished herein. For example, high levels of circulating miR-25, -22, -19a, -19b, -210, and -93, and low levels of miR199a-3p may mark resistance to a doxorubicin-cyclophosphamide regimens paired with either cisplatin or paclitaxel, as suggested by the network analyses illustrated in Figure 3. Further empirical and clinical evidence for these suggested associations will be required to confirm specific circulating markers.

Meta-analysis of survival in breast cancer patients indicated that miR-93, miR-210, miR-19a, and miR-19b are particularly strong candidates as markers based on their association with poor survival. While this analysis was done across breast cancer subtypes, and without regard to specific chemotherapeutic regimens, it does have general implications on the association of these circulating markers with response to therapy for breast cancer. 

There is abundant literature supporting roles for the targeted pathways we have identified in resistance to chemotherapy. Many anticancer drugs, such as DNA crosslinking agents, alkylating agents, and TKIs act by inhibiting proliferation and inducing apoptosis in proliferating tumor cells. MiRNAs can regulate drug resistance by regulating key proliferation and cell cycle genes. Souza et al. demonstrated that the expression of miRNA-25-3p was elevated in TNBC patient serum in comparison with matched healthy controls [38]. MiR-25-3p promotes proliferation in several tumor types, and miR-25-3p suppression can induce apoptosis [39]. The biological mechanism involved in miR-25-3p regulation of TNBC is not yet known. One possible mechanism is the targeting of B-cell translocation gene 2 (BTG2) [39]. Depletion of BTG2 via miR-25-3p may lead to AKT and MAPK signaling activation, which are intimately involved in cell proliferation and apoptosis. BTG2 is a negative regulator of these pathways. The specific mechanisms underlying the activation of these pathways in TNBC remain poorly understood. 

PTEN is an inhibitor of the PI3K/Akt signaling pathway and an inhibitor of CDK that is associated with cisplatin chemoresistance and metastasis [40]. PI3K/PTEN/AKT pathway alterations, involving mutations of either PIK3CA, PTEN, or AKT1, are present in 25% of TNBC [41]. The PTEN tumor suppressor has been shown to be negatively regulated by miRNA binding within its 3′-UTR [42]. MiR-93, one of the significant miRNAs in our study, was found to target PTEN in TNBC cells and upregulate drug resistance [28,29]. Our identification of mirR-93 in the circulation of TNBC patients extends previous findings of increased miR-93 expression in TNBC tumor tissues [29,30]. Functional analysis confirmed a role of miR-93 in chemoresistance to cisplatin. MiR-93 has been implicated in the onset, progression, and metastases of TNBC. MiR-93-3p expression was found to be significantly higher in TNBC than in other breast cancer subtypes, implicating miR-93 as a potential biomarker related to the biological and clinical characteristics of TNBC [43,44,45]. Li et al. found that miR-105 and miR-93-3p levels were increased in TNBC and correlated with poor survival [46]. The authors found that these miRNAs activated Wnt/β-catenin signaling through the targeting of SFRP1, which promoted chemoresistance, progression, and stemness. MiRNAs have been shown to target PTEN, to promote the expression of CDK and modify chemoresistance in cancer [47]. mTOR is involved in the PI3K/Akt signaling pathway, promoting proliferation and survival. miR-199a-3p, which we found to be significantly downregulated in TNBC, can target mTOR and c-Met, affecting sensitivity to doxorubicin [48]. G(1)-phase cell cycle arrest occurred upon reconstitution of miR-199a-3p expression, resulting in reduced invasion and increased Dox-induced apoptosis. 

P53-dependent cell cycle G1 phase arrest is mediated by CDKN1A/P21 in response to stress stimuli [49]. CDKN1A inhibits CDK2 and CDK1, regulating cell cycle progression at the G1 and S phase [50]. Induction of aberrant G1-S transition and promotion of cell proliferation and tumorigenesis through miRNA targeting of CDKN1A has been demonstrated. CDKN1A can also interact with PCNA and regulate DNA damage repair and DNA replication [51]. A study in NPC (nasopharyngeal carcinoma) showed that miR-93 was upregulated in tissues and that re-expression of miR-93 promoted cell growth in vitro [52]. The 3′-UTR of CDKN1A was targeted by miR-93, leading to its degradation. Several studies have identified other miR-93 target genes, including RAB11 family genes and PTEN [52]. Cumulatively, these findings indicate that miR-93 plays a key role in cancer progression.

EMT and stemness can be involved in resistance to chemotherapy and targeted therapies in TNBC [12]. EMT- and stemness-related mechanisms of chemoresistance have been controversial, but may involve quiescence and immune evasion [40,41]. EMT can be promoted by miR-22 in breast cancer cells with increased invasiveness and metastasis, making this miRNA a potential therapeutic target [53,54,55]. MiR-19a expression can be increased by TNF-α and is associated with lymph node metastasis and mesenchymal markers, suggesting a transition to the metastasis promoting EMT [56]. miR-19a and miR-22 were previously associated with carcinogenesis, with multiple effects on proliferation, invasion, EMT, and metastasis in HER2 positive breast cancer [34]. MiR-93 has been shown to cause stemness, cisplatin resistance, and metastasis trough Wnt–β-catenin signaling in TNBC [12]. MiR-93 has been implicated as a potential immune regulator in breast cancer cells through targeting programmed death-ligand 1 (PD-L1)/cluster of differentiation 274 (CD274), programmed Cell Death 1 Ligand 2 (PDCD1LG2), and natural killer cell cytotoxicity receptor 3 ligand 1 (NCR3LG1) [42]. We identified miR-93 as up-regulated in TNBC compared to luminal breast cancers. These miRNAs have been associated with tumorigenesis and the progression of cancers [28,39]. 

Altogether, our findings, in the context of the published literature on miRNAs in TNBC, indicate that the circulating miRNAs we have identified are suitable candidates as markers in TNBC, with a high potential to improve personalized clinical approaches to this disease. We presented a well-defined subset of significantly aberrant circulating miRNAs, with correlations to clinical outcome and well-founded evidence for roles in breast cancer drug resistance pathways. These results have implications for therapeutic targeting of these aberrantly expressed miRNAs, which may be key in disease pathophysiology and drug response. Our illustration of the associations of these miRNAs with pathways regulating response to specific drugs opens the possibility for targeting these miRNAs in combination with drugs that may be synergistically effective. Further studies exploring the mechanistic links of these miRNAs to drug resistance and their predictive value for response to therapy are justified.

We recognize certain limitations to this study, including the small number of differentially expressed miRNAs identified. Due to this limitation, the complete delineation and evaluation of markers was difficult, and large scale studies are necessary to confirm these results before clinical practice. Future experiments should confirm findings in plasma and track these markers through the course of treatment. Despite this limitation, the current study identified potential TNBC specific circulating markers related to chemoresistance and illustrated the utility of the multi-dimensional analyses described herein to identify potential markers and mechanisms of chemoresistance in TNBC. In-depth mechanistic studies of the miRNA–gene network connections to chemoresistance and clinical outcome of TNBC delineated herein are outside the scope of the current study but would be justified in future research.

## 5. Conclusions

In the current study, we identified significant differentially expressed circulating miRNAs in TNBC patients (miR-19a-3p, miR-19b-3p, miR-25-3p, miR-22-3p, miR-210-3p, miR-93-5p, and miR-199a-3p) that regulate several molecular pathways (PAM(PI3K/Akt, mTOR), HIF-1, TNF, FoxO, Wnt, and JAK/STAT, TKIs, PD-1/PD-L1) involved in chemoresistance. Integrated analysis of multi-dimensional data revealed potential mechanisms of drug-resistance related to circulating miRNAs in TNBC. With further correlative and mechanistic studies, these may prove to be useful as non-invasively accessible markers of drug response and resistance in TNBC. Our findings show that up-regulated miRNAs such as these may be suitable candidates for theranostic markers and implicate prominent molecular pathways of resistance in TNBC. In addition, investigation of the clinical and the biological aspects of TNBC at the molecular level may have long term clinical implications and help clinicians in treatment, decision-making, and better post-treatment care in TNBC. Our findings may direct future clinical research in the field of TNBC theranostic markers. 

## Figures and Tables

**Table 1:** Patient Characteristics.

Parameter/Feature		Breast Cancer (n = 93)	Healthy Control (n = 34)	*p*-Value (Chi-Squared Test)
Age (Mean Years ±SD)		46 ± 10.55	29 ± 7.5	
	≤35	16 (17.20%)	27 (79%)	2.19 × 10^−10^
	>35	77 (82%)	7 (20%)	
	<25	12 (12.90%)	5 (14.7%)	
	BMI	25–29.9	35 (37.63%)	10 (29.4%)	
30–34.9		24 (25.81%)	12 (35.3%)	0.4732
35–39.9		10 (10.75%)	7(20.5%)	
≥40		11.83%)	-	
Missing		1 (1.08%)	-	
Histology	*IDC	84 (90.32%)		
	*ILC	8 (8.62%)		
	Metaplastic	1 (1.08%)		
	2	28 (30.11%)		
	3	58 (62.37%)		
	Missing	7 (7.53%)		
Subtype	TNBC	36 (38.71%)		
	Luminal A	16 (17.20%)		
	Luminal B	41 (44.09%)		
ER status	positive	55 (59.14%)		
	negative	38 (40.86%)		
PR status	positive	54 (58.06%)		
	negative	39 (41.94%)		
HER2	positive	16 (17.2%)		
	negative	77 (82.8%)		
Tumor size	≤2.0 cm	19 (20.43%)		
	2.1 cm–5.0 cm	38 (40.86%)		
	>5.0 cm	11 (11.83%)		
	Missing	25 (26.88%)		
Metastasis status	M0	70 (75.27%)		
	M1	21 (22.58%)		
	Mx	2 (2.15%)		
Lymph node	positive	54 (58.06%)		
	negative	34 (36.56%)		
	Missing	5 (5.38%)		
Ki67	≤15	20 (21.51%)		
	>15	73 (78.5%)		

*IDC, invasive ductal carcinoma; *ILC, invasive lobular carcinoma; BMI, body mass index; ER, estrogen receptor; PR, progesterone receptor; SD, standard deviation; TNBC, triple-negative breast cancer.

**Table 2:** Top significant unique KEGG pathways enriched in TNBC at the 3′-UTR, corresponding to the significant terms of adjusted *p*-value (BH) ≤ 0.05.

KEGG IDs	KEGG Terms	Top Hits	*p*-Value	Adjusted *p*-Value (BH)
hsa04151	PI3K-Akt signaling pathway	47	0	0
hsa04110	Cell cycle	23	0	0
hsa01521	EGFR tyrosine kinase inhibitor resistance	21	0	0
hsa04218	Cellular senescence	31	0	0
hsa04140	Autophagy	24	0	0
hsa04917	Prolactin signaling pathway	17	0	0
hsa04115	p53 signaling pathway	18	0	0
hsa04066	HIF-1 signaling pathway	20	0.0001	0.0006
hsa04668	TNF signaling pathway	20	0.0001	0.0006
hsa01522	Endocrine resistance	18	0.0002	0.001
hsa04210	Apoptosis	22	0.0002	0.001
hsa04014	Ras signaling pathway	31	0.0003	0.0015
hsa05235	PD-L1 expression and PD-1 checkpoint pathway in cancer	16	0.0005	0.0023
hsa04010	MAPK signaling pathway	36	0.0005	0.0023
hsa04068	FoxO signaling pathway	20	0.0008	0.0033
hsa01524	Platinum drug resistance	13	0.0018	0.0064
hsa04137	Mitophagy	12	0.002	0.0069
hsa04012	ErbB signaling pathway	14	0.0023	0.0078
hsa04370	VEGF signaling pathway	11	0.0029	0.0097
hsa04150	mTOR signaling pathway	18	0.0162	0.0352
hsa04310	Wnt signaling pathway	18	0.021	0.0426
hsa04630	JAK-STAT signaling pathway	21	0.0034	0.0112
hsa04350	TGF-beta signaling pathway	14	0.0052	0.0154
hsa04152	AMPK signaling pathway	16	0.0076	0.0206
hsa04390	Hippo signaling pathway	18	0.018	0.0387
hsa04662	B cell receptor signaling	11	0.0229	0.0446
hsa04660	T cell receptor signaling	13	0.0229	0.0446

BH, Benjamini–Hochberg; KEGG, Kyoto Encyclopedia of Genes and Genomes.

**Table 3:** The most significant gene ontology terms of predicted miRNA target genes. Significant unique gene ontology terms for biological processes enriched in TNBC corresponding to the significant terms of adjusted *p*-value (BH) ≤ 0.05.

GO-BP IDs	GO-BP Terms	Top Terms/Hits	*p*-Value	Adjusted *p*-Value (BH)
GO:0007049	Cell cycle	36	0	0
GO:0043066	Negative regulation of apoptotic process	56	0	0
GO:0006974	Cellular response to DNA damage stimulus	33	0	0
GO:0051301	Cell division	40	0.0001	0.007
GO:0001934	Positive regulation of protein phosphorylation	23	0.0002	0.0123
GO:0071456	Cellular response to hypoxia	19	0.0003	0.0147
GO:0016567	Protein ubiquitination	47	0.0003	0.0147
GO:0070317	Negative regulation of G0 -G1 transition	10	0.0004	0.0179
GO:0030177	Positive regulation of Wnt signaling pathway	9	0.0007	0.0264
GO:0000082	G1-S transition of mitotic cell cycle	16	0.0007	0.0264
GO:0019221	Cytokine mediated signaling pathway	32	0.0009	0.0276
GO:0035019	Somatic stem cell population_ maintenance	12	0.0009	0.0276
GO:0048147	Negative regulation of fibroblast proliferation	8	0.0008	0.0276
GO:0006470	Protein de-phosphorylation	20	0.001	0.0289
GO:0010628	Positive regulation of gene expression	40	0.0011	0.03
GO:0006606	Protein import into nucleus	13	0.0015	0.0351
GO:0016055	Wnt signaling pathway	23	0.0015	0.0351
GO:0042149	Cellular response to glucose starvation	9	0.0019	0.0389
GO:0016579	Protein de-ubiquitination	29	0.0021	0.0397
GO:0031647	Regulation of protein stability	13	0.0022	0.04
GO:0045787	Positive regulation of cell cycle	8	0.0023	0.0403
GO:0001933	Negative regulation of protein phosphorylation	12	0.0026	0.044
GO:0032467	Positive regulation of cytokinesis	8	0.0041	0.0516
GO:0000079	Regulation of cyclin-dependent protein serine-threonine kinase activity	10	0.0035	0.0516
GO:0071560	Cellular response to transforming growth factor beta stimulus	10	0.0039	0.0516
GO:0050821	Protein stabilization	21	0.004	0.0516
GO:0001836	Release of cytochrome c from mitochondria	6	0.0039	0.0516
GO:0014068	Positive regulation of PI3K signaling	13	0.0038	0.0516
GO:0044772	Mitotic cell cycle phase transition	6	0.0047	0.0537
GO:0010629	Negative regulation of gene expression	25	0.0046	0.0537
GO:1902895	Positive regulation of pri-miRNA transcription by RNA polymerase II	7	0.0045	0.0537
GO:0045737	Positive regulation of cyclin-dependent protein serine-threonine kinase activity	7	0.0045	0.0537
GO:0006366	Transcription by RNA polymerase II	28	0.005	0.0558
GO:0071230	Cellular response to amino acid stimulus	9	0.0058	0.0565
GO:0014065	PI3K signaling	7	0.0061	0.0565
GO:0061418	Regulation of transcription from RNA polymerase II promoter in response to hypoxia	11	0.0058	0.0565
GO:0010507	Negative regulation of autophagy	9	0.0052	0.0565
GO:0007265	Ras protein signal transduction	11	0.0063	0.0573
GO:0050680	Negative regulation of epithelial cell proliferation	10	0.0065	0.058

**Table 4:** Significant TNBC miRNAs and their respective target genes associated with TNBC drugs.

TNBC Drugs	miRNAs ID	Target Genes
5-fluoroucil	miR-19a;miR-19b;miR-25;miR-199a-3p; miR-22;miR-93	NFKB1; BCL2; PTEN; MSH2
fluorouracil	miR-19a;miR-19b;miR-25;miR-199a-3p; miR-22;miR-93	ABCB1;GSTT1;ABCC4;NFKB1;UGT1A1;RRM2;ABCG2;ERBB2;IGF1;IGF2;IGFBP3;TP53;BCL2;CDKN1A;ABCC3;SMUG1;TDG;MBD4;ABCC5;UPP1;UPP2;PTEN;UCK2;CLCN6;WDR7;SLC35A2;APC;RUNX3;FXYD3;FDXR;DUT;DHFR;UMPS;MTHFR;DPYD;TPMT;UPB1;FASLG;ERCC1;NOS3;GNAS;CES2;TK1;XRCC3;NT5C;GSTM1;CYP2A6;SLC19A1;KLC3;UNG
gefitinib	miR-19a;miR-19b;miR-25;miR-199a-3p; miR-22;miR-210;miR-93	ABCB1;CYP3A4;PTGS2;UGT1A1;CYP1A1;ABCG2;CCND1;ABL1;APAF1;IL15;KIT;PDGFRB;ERBB2;EGF;ERBB3;IL8;IL8RA;GAB1;MET;EMP1;CYP2C9;AKT1;FUS;EGFR
gemcitabine	miR-19a;miR-19b;miR-25;miR-199a-3p; miR-22;miR-210;miR-93	ABCB1;ABCC4;RRM2;ABCG2;ERBB2;BCL2;CDKN1A;SP1;PRKCA;PRKCE;XRCC5;ABCC3;ERBB3;PARP1;AICDA;ABCC5;PTEN;TOP2A;HPRT1;NT5C2;MKI67;EPC2;CLU;POLE;GPM6A;IQGAP2;TGM3;VAV3;BCL2L1;DCK;CDKN1B;ATP7B;ERCC1;POLS;USF2;DCTD;SLC28A1;AKT1;POLA2;PRKCB1;CDKN2A;SLC29A2;IGFBP1;USF1;VEGFA;SLC28A2;CMPK1;EGFR;BAX
bevacizumab	miR-19a;miR-19b;miR-25;miR-199a-3p; miR-22;miR-210	ABCB1;UGT1A1;IGF1;IGF2;IGFBP3;KDR;HIF1A;VHL;DPYD;FCGR2A;FCGR3A;GSTM1
alkylating agents	miR-19a;miR-19b;miR-25;miR-199a-3p; miR-22;miR-210;miR-93	MDM2;MTHFR
capecitabine	miR-19a;miR-19b;miR-25;miR-199a-3p; miR-22;miR-210;miR-93	GSTT1;PTGS2;UGT1A1;RRM2;FRAP1;UPP1;UPP2;ERCC6;GSTT1;PTGS2;UGT1A1;RRM2;FRAP1;UPP1;UPP2;ERCC6;MTHFR;CYP2C9;UGT1A1;FRAP1;DPYD;GSTA1;UPB1;ERCC1;MTHFR;UGT1A1;DCTD;FRAP1;DPYD;CES2;TK1;GSTM1;MTHFR;CYP2C9;UGT1A1;DCTD;DPYD;GSTT1;MTHFR;FRAP1;GSTT1;PTGS2;VEGFA;MTHFR;RRM2;FRAP1;DPYD;CES2;GSTA1;UPP1;UPP2;APEX2;RAD54B
carboplatin	miR-19a;miR-19b;miR-25;miR-199a-3p; miR-22;miR-210;miR-93	CYP3A4;SLC22A2;MTHFR;ABCG2;ATP7A;DPYD;TP53;CDKN1A;CAMTA1;CYP1B1;MAPT;ABCB1;UGT1A1;CYP2C8;ABCC1;GSTM1;SLC19A1
cisplatin	miR-19a;miR-19b;miR-25;miR-199a-3p; miR-22;miR-210;miR-199a;miR-93	GSTT1;VEGFA;DHFR;SLC22A2;ABCC4;NQO1;MDM2;RRM2;ABCG2;ATP7A;GSTM4;GCLC;GCLM;GPX6;GNAS;ABL1;APAF1;FUS;KIT;PDGFRB;FRAP1;AKT1;MCL1;ERBB2;EGFR;DPYD;TPMT;XPC;CDKN2A;TP53;BCL2;BCL2L1;XIAP;DCK;CDKN1A;RB1;ERBB3;GSTA1;ABCC5;SLC29A2;PTEN;TOP2A;LRP2;SLC31A1;HPRT1;NT5C2;ATP7B;BAX;BID;SUMO1;CD3EAP;ATM;DNAJC15;MKI67;GSTM3;GJA1;BRCA2;EPHA2;XRCC2;TWIST1;ATP8B4;CDKN2D;EBF3;FAM57A;FCHSD1;IRF2BP2;LRRC32;MYO5B;NBEAL2;PARD6B;PGM1;PQLC3;SHMT2;SLC6A8;SORBS2;STK17A;CDK6;ABCB1;SOCS3;TOP2B;CYP2E1;UGT1A1;GPX7;IL15;XRCC5;ABCC3;PPP1R13L;HOXB9;CSF1;UMPS;SLCO1B1;GSTA4;TOP1;GATM;ARVCF;ERCC1;BAK1;DDIT4;NEK2;PFKFB4;ABCC1;GPX2;UBE2I;GALNTL4;XPA;GSTM1;GPX1;GPX3;GSTM2;GSTM5;ALDH7A1;NUF2;TMEM37;IGFBP1;CD44
cetuximab	miR-19a;miR-19b;miR-25;miR-199a-3p; miR-22;miR-210;miR-93	PTGS2;VEGFA;CCND1;EGFR;KRAS;FCGR3A;IL8;HBEGF;EGF;IL8RA;FCGR2A
cyclophosphamide	miR-19a;miR-19b;miR-25;miR-199a-3p; miR-22;miR-210;miR-93	CYP3A4;GSTT1;MTHFR;DRD2;CYP1A2;ABCG2;NR1I2;ERBB2;SOD2;TP53;BCL2;CDKN1A;GSTA1;CYP1B1;CD3EAP;GSTM3;WDR7;ABCB1;VDR;CYP2E1;UGT1A1;PPP1R13L;CYP2B6;CYP2C9;CYP2C8;NR1I3;ERCC1;NOS3;GSTM1;CYP2A6
dexamethasone	miR-19a;miR-19b;miR-25;miR-199a-3p; miR-22;miR-210;miR-93	ABCB1;CYP3A4;GSTT1;PTGS2;TNF;VDR;CREBBP;EP300;AGT;CYP2E1;UGT1A1;ADRB2;CORIN;NR1I2;PIK3CA;TGFBR2;PDPK1;NR3C1;GNB1;MAP2K3;MAPK14;MYD88;TLR2;PIK3R1;IL1A;BDKRB2;IL8;IL3;SMARCD1;MAP4K4;TGFBR1;CAV1;SMAD3;SMAD4;ACTB;ARID1A;NF1;SMARCC1;CYP2B6;MTHFR;CYP1A2;CYP2C9;CYP2C8;DUSP1;TPMT;GTF2A1;GTF2E1;POLR2A;IKBKG;IL13;NOS3;GNAS;MAP2K6;SMARCE1;MAPK11;GTF2B;SMARCA4;SMARCC2;GSTM1;CYP2A6;AKT1;NPPA;MAP3K7;SLC19A1;TGFB3;IL5;IL6;IL10;CYP3A43
docetaxel	miR-19a;miR-19b;miR-25;miR-199a-3p; miR-22;miR-210;miR-93	ABCB1;CYP3A4;CYP2E1;ABCG2;ATP7A;SLC10A2;SPG7;PPARD;TNFAIP2;APAF1;NR1I2;ERBB2;IGF2;KRAS;PIK3CA;TGFBR2;TGFBR3;XRCC4;CYP2F1;EGF;TP53;BCL2;CDKN1A;ABCC5;PTEN;PLK1;CYP1B1;MAPT;IGFBP2;WDR7;BRCA2;CYP2B6;MTHFR;CYP2C9;CYP2C8;CHST3;GSTA4;DPYD;TPMT;CYP2C18;BCL2L1;RPN2;CDKN1B;ATP7B;MFAP4;ABCC1;RPL13;TMEM43;GSTM1;CYP2A6;GSTM5;IGF2AS;ABCC6;IGFBP1;SLCO1B3;NAT2;EGFR;XPC
doxorubicin	miR-19a;miR-19b;miR-25;miR-199a-3p; miR-22;miR-210;miR-93	MTHFR;HRH1;NFKB1;NQO1;XDH;CAT;NOS1;CYCS;ABCG2;NR1I2;AKT1;ERBB2;SETD4;TP53;BCL2;RALBP1;HIF1A;GSTA1;CASP3;ABCC5;PTEN;TOP2A;FOXO3;CYP1B1;WDR7;MET;ERBB4;SLC19A3;ABCB1;CBR1;TOP2B;PLK1;PPP2R4;MMP1;CYP2C8;SOD1;PIM1;BAK1;OXTR;CYBA;NOS3;ABCC1;MTOR;GSTM1;AKR1A1;GPX1;SCN5A;CD44
epirubicin	miR-19a;miR-19b;miR-25;miR-199a-3p; miR-22;miR-210;miR-93	ABCB1;ERBB2;SOD2;TOP2A;NQO1;ABCC1
everolimus	miR-19a;miR-19b;miR-25;miR-199a-3p; miR-22;miR-210;miR-93	FRAP1;MKI67
fulvestrant	miR-19a;miR-19b;miR-25;miR-199a-3p; miR-22;miR-93	ESR1;ERBB2;ADORA1
letrozole	miR-19a;miR-19b;miR-25;miR-199a-3p; miR-22;miR-210;miR-93	CCND1;COLEC12;CTSK;DKK3;EGR1;GPNMB;KIAA0101;PHLDA2;CYP19A1;COL3A1;DCN;DUSP1;IRS1;SFRP4;CCNB1;MMP2;ZWINT;CYR61;HMGB2;MLF1IP;NUSAP1;SERPINA3;GEM;TPBG
metformin	miR-19a;miR-19b;miR-25;miR-199a-3p; miR-22;miR-210;miR-93	ABCB1;SLC22A3;ABCG2;CCND1;ERBB2;SREBF1;RPS6KB1;CDKN1A;PRKAA1;PRKAA2;PRKAB2;STK11;SLC22A2;CYP2C9;CDKN1B;PRKAB1;PRKAG2;NDUFA1;NDUFS1;NDUFS4;SLC47A2
methotrexate	miR-19a;miR-19b;miR-25;miR-199a-3p; miR-22;miR-210;miR-93	ABCB1;CYP3A4;GSTT1;TNF;SLCO1A2;ABCC4;VDR;UGT1A1;ADRB2;SLCO4C1;ABCG2;ERBB2;MTRR;TP53;ITGB2;RALBP1;PTPRC;SPP1;NR3C1;CDKN1A;TERF1;ABCC3;CREB1;IL8RB;MTR;RFC1;IL8RA;ITGAL;ADA;HPRT1;NP;UCK2;ADORA2A;GART;ARID5B;SLC16A7;ELMO1;CLCN6;GJA1;MLL;HHEX;FTH1;GCH1;ABLIM1;ACP2;ANKRD12;AP2B1;ARF4;ARHGAP5;ARL4C;ATF1;ATRN;CA3;CLK1;CNOT8;CP;CTSL1;CUL4B;DPYSL2;DSG1;EFNB2;ETV5;FRK;FZD6;GOLGA2;GPR137B;H3F3B;HIC2;IAPP;IL1R1;JRKL;KIAA0143;KIAA1467;KIF3A;MAN2B2;MPHOSPH9;MYH15;NMT1;PARG;PCDH9;PLS1;POLE;PPP1CB;PRDM2;PRKY;PTPRG;RAB31;RAPH1;RBBP8;SACS;SAP18;SMCHD1;SS18;ST18;STK24;STK38;TGIF;TXK;WIF1;CD97;CHST1;IGFBP4;TMEM45A;DHFR;CYP2B6;MTHFR;ADORA1;XDH;SLCO1B1;DPYD;TPMT;JUN;TLR4;RB1;MSH3;SLC22A9;ITGAX;NT5E;PPAT;FAM3C;FGF9;MTHFD2;CRYZ;CSH2;GALNT7;GDF11;GZMM;HAT1;IL1RL1;MRPL33;MYLK;NUP98;PRKCQ;RDX;TESK1;TOB2;YY1;NOS3;ABCC1;TK1;MAX;ADORA3;ADORA2B;AP1S2;IL1RN;NRXN2;RAB5C;RCC1;SSX1;GSTM1;G6PD;ABCC11;SLC22A11;SLC19A1;GBF1;MPO;ITPA;AMT;ADRA1D;CST7;DEFA4;GBE1;GCHFR;GDF10;LCN2;PTK7;FOLR1;PECAM1;PTS;SLC22A8;SLCO1B3;NAT2;APP;AHCY;EIF4A1;BAX;E2F1;BYSL;FZD2;GNG10;PF4V1;PRG1;RNASE6;TCEB3
paclitaxel	miR-19a;miR-19b;miR-25;miR-199a-3p; miR-22;miR-210;miR-93	ABCB1;CYP3A4;ABCG2;NR1I2;ERBB2;TP53;BCL2;CDKN1A;CASP3;FOXO3;CYP1B1;AKT2;MAPT;WDR7;APC;CYP2B6;MTHFR;CYP1A2;CYP2C8;SLCO1B1;DPYD;BAK1;ABCC1;PHB;GSTM1;CYP2A6;VEGFA;SLCO1B3; CD44
Platinum/platinum compounds	miR-19a;miR-19b;miR-25;miR-199a-3p; miR-22;miR-210;miR-93	ATP7A;ATP7B;SLC22A3;SLC22A2
taxol	miR-19a;miR-19b;miR-25;miR-199a-3p; miR-22;miR-210;miR-93	PTEN;CSF1
topotecan	miR-19a;miR-19b;miR-25;miR-199a-3p; miR-22;miR-210;miR-93	ABCB1;CYP3A4;ABCG2;NR1I2;ERBB2;TP53;BCL2;PTEN;WDR7;MTHFR
vincristine	miR-19a;miR-19b;miR-25;miR-199a-3p; miR-22;miR-210;miR-93	ABCB1;CYP3A4;GSTT1;VDR;UGT1A1;ABCG2;NR1I2;BCL2;NR3C1;AAK1;MTHFR;TPMT;DCK;GSTA1;ABCC1;FLT3;NPM1;GSTM1;SLC19A1;ABCC6;XIAP
olaparib	miR-93;miR-19a;miR-19b;miR-199a-3p; miR-22	PTGS2;PARP1;KDR;CCR4

## Data Availability

The data presented in this study are available in the article.

## Supplementary Materials

The following are available online at https://www.mdpi.com/article/10.3390/genes12040549/s1, Table S1: List of circulating miRNAs in the plasma with the appropriate log2 fold change (FC) and Adjusted *p*-value (BH), Figure S1: Expression pattern of miRNAs in the TCGA-BRCA cohort, Figure S2. Hierarchical clustering and heatmap of miRNAs across samples shows the expression pattern of significant differentially expressed circulating miRNAs. Figure S3. Whisker plots of comparisons between miRNA expression levels in different subtypes of breast cancer and normal healthy profiled from TCGA. Table S2. Circulating miRNAs in the plasma that were differentially expressed in healthy control and breast cancer patients, with fold change (FC), *p*-value and Adjusted *p*-value (BH) < 0.05. Figure S4. Whisker plots of comparisons between miRNA expression levels in normal control and triple negative breast cancer patients samples (TNBC). Figure S5. AUC/ROC analysis (Receiver operating characteristic curve and area under the ROC Curve) of significant differentially regulated miRNAs. Figure S6. Enriched KEGG signaling pathways in TNBC at the 3′-UTR of target genes with adjusted *p*-values Figure S7. Enriched signaling pathways by Kyoto Encyclopedia of Genes and Genomes (KEGG) among triple negative breast cancer (TNBC) arranged by 3′-UTR, 5'-UTR and CDS region targeting shown as a circular bar-chart including significant signaling pathways. Figure S8. Gene ontologies enriched in TNBC is shown as circular bar chart. Figure S9: Gene ontologies enriched in Luminal breast cancer tumors are shown as circular bar chart, Figure S10: GO significant biological process (GO-BP) enriched in TNBC, Figure S11: PI3K-AKT pathway in TNBC, Figure S12: Cell cycle pathways, Supp. Figure S13: EGFR tyrosine kinases inhibitor resistance (TKIs) pathway in TNBC, Figure S14: Cellular senescence pathway, Figure S15: PD-1/PD-L1 pathway, Figure S16: mTOR signaling pathway, Figure S17: JAK/STAT signaling pathway, Figure S18: Hierarchical Clusters of miRNAs/KEGG targeted pathways.
