# Supplementary material for: Clinical Identification of Dysregulated Circulating microRNAs and Their Implication in Drug Response in Triple Negative Breast Cancer (TNBC) by Target Gene Network and Meta-Analysis
Source: Genes (Basel). 2021 Apr 9;12(4):549. doi: 10.3390/genes12040549 (PMC8068962; doi:10.3390/genes12040549)
Supplement: Supplementary file 1 [file genes-12-00549-s001.pdf]

All Breast cancer vs Normal			TNBC vs Normal			Luminal vs Normal			TNBC vs Luminal		
miRID	log2 Fold Change	BH Adjusted Pvalue	miRID	log2 Fold Change	BH Adjusted Pvalue	miRID	log2 Fold Change	BH Adjusted Pvalue	miRID	log2 Fold Change	BH Adjusted Pvalue
hsa-let-7a-5p	0.58	0.08	hsa-let-7a-5p	0.44	0.23	hsa-let-7a-5p	0.66	0.06	hsa-let-7a-5p	-0.29	0.66
hsa-let-7b-5p	1.16	0.00	hsa-let-7b-5p	1.10	0.00	hsa-let-7b-5p	1.20	0.00	hsa-let-7b-5p	-0.18	0.69
hsa-let-7c-5p	0.89	0.00	hsa-let-7c-5p	0.78	0.03	hsa-let-7c-5p	0.97	0.00	hsa-let-7c-5p	-0.26	0.66
hsa-let-7d-5p	0.49	0.14	hsa-let-7d-5p	0.46	0.21	hsa-let-7d-5p	0.50	0.22	hsa-let-7d-5p	-0.10	0.81
hsa-let-7e-5p	0.28	0.44	hsa-let-7e-5p	0.18	0.63	hsa-let-7e-5p	0.34	0.42	hsa-let-7e-5p	-0.22	0.66
hsa-let-7f-5p	-0.07	0.83	hsa-let-7f-5p	-0.17	0.66	hsa-let-7f-5p	0.00	0.99	hsa-let-7f-5p	-0.23	0.66
hsa-let-7g-5p	-0.20	0.69	hsa-let-7g-5p	-0.05	0.91	hsa-let-7g-5p	-0.29	0.61	hsa-let-7g-5p	0.22	0.69
hsa-let-7i-5p	0.72	0.00	hsa-let-7i-5p	0.77	0.01	hsa-let-7i-5p	0.68	0.01	hsa-let-7i-5p	0.03	0.92
hsa-miR-100-5p	0.69	0.25	hsa-miR-100-5p	0.23	0.62	hsa-miR-100-5p	0.98	0.35	hsa-miR-100-5p	-0.82	0.66
hsa-miR-125b-5p	0.71	0.23	hsa-miR-125b-5p	0.17	0.63	hsa-miR-125b-5p	1.05	0.33	hsa-miR-125b-5p	-0.96	0.61
hsa-miR-128-3p	0.12	0.83	hsa-miR-128-3p	-0.25	0.58	hsa-miR-128-3p	0.35	0.91	hsa-miR-128-3p	-0.67	0.66
hsa-miR-130a-3p	0.38	0.17	hsa-miR-130a-3p	0.54	0.09	hsa-miR-130a-3p	0.27	0.45	hsa-miR-130a-3p	0.26	0.66
hsa-miR-130b-3p	0.43	0.11	hsa-miR-130b-3p	0.53	0.09	hsa-miR-130b-3p	0.37	0.26	hsa-miR-130b-3p	0.13	0.71
hsa-miR-132-3p	0.95	0.12	hsa-miR-132-3p	0.44	0.20	hsa-miR-132-3p	1.26	0.25	hsa-miR-132-3p	-0.88	0.66
hsa-miR-140-5p	0.24	0.80	hsa-miR-140-5p	0.19	0.63	hsa-miR-140-5p	0.27	0.94	hsa-miR-140-5p	-0.18	0.89
hsa-miR-145-5p	-0.19	0.83	hsa-miR-145-5p	-0.25	0.63	hsa-miR-145-5p	-0.16	0.99	hsa-miR-145-5p	-0.13	0.92
hsa-miR-148a-3p	0.56	0.04	hsa-miR-148a-3p	0.54	0.08	hsa-miR-148a-3p	0.56	0.06	hsa-miR-148a-3p	-0.05	0.89
hsa-miR-152-3p	-0.19	0.83	hsa-miR-152-3p	-0.67	0.09	hsa-miR-152-3p	0.11	0.99	hsa-miR-152-3p	-0.83	0.66
hsa-miR-15a-5p	0.68	0.25	hsa-miR-15a-5p	1.01	0.01	hsa-miR-15a-5p	0.48	0.72	hsa-miR-15a-5p	0.51	0.69
hsa-miR-15b-5p	0.06	0.83	hsa-miR-15b-5p	0.09	0.79	hsa-miR-15b-5p	0.05	0.99	hsa-miR-15b-5p	-0.01	0.97
hsa-miR-16-5p	0.90	0.00	hsa-miR-16-5p	1.14	0.00	hsa-miR-16-5p	0.75	0.01	hsa-miR-16-5p	0.33	0.45
hsa-miR-17-5p	-0.06	0.83	hsa-miR-17-5p	0.38	0.31	hsa-miR-17-5p	-0.33	0.45	hsa-miR-17-5p	0.63	0.23
hsa-miR-181a-5p	-1.47	0.56	hsa-miR-181a-5p	-2.59	0.15	hsa-miR-181a-5p	-0.76	0.93	hsa-miR-181a-5p	-1.89	0.55
hsa-miR-181b-5p	0.11	0.80	hsa-miR-181b-5p	0.16	0.64	hsa-miR-181b-5p	0.08	0.97	hsa-miR-181b-5p	0.07	0.89
hsa-miR-186-5p	0.23	0.60	hsa-miR-186-5p	0.61	0.09	hsa-miR-186-5p	-0.02	0.99	hsa-miR-186-5p	0.56	0.23
hsa-miR-18a-5p	0.30	0.40	hsa-miR-18a-5p	0.66	0.07	hsa-miR-18a-5p	0.07	0.99	hsa-miR-18a-5p	0.53	0.26
hsa-miR-195-5p	0.75	0.00	hsa-miR-195-5p	0.88	0.00	hsa-miR-195-5p	0.67	0.01	hsa-miR-195-5p	0.13	0.71
hsa-miR-199a-3p	-1.49	0.00	hsa-miR-199a-3p	-1.49	0.00	hsa-miR-199a-3p	-1.49	0.00	hsa-miR-199a-3p	-0.04	0.94
hsa-miR-199a-5p	-1.04	0.61	hsa-miR-199a-5p	-0.51	0.79	hsa-miR-199a-5p	-1.38	0.58	hsa-miR-199a-5p	0.80	0.79
hsa-miR-19a-3p	0.56	0.04	hsa-miR-19a-3p	0.86	0.00	hsa-miR-19a-3p	0.37	0.30	hsa-miR-19a-3p	0.42	0.29
hsa-miR-19b-3p	0.56	0.04	hsa-miR-19b-3p	0.90	0.00	hsa-miR-19b-3p	0.35	0.33	hsa-miR-19b-3p	0.49	0.23
hsa-miR-205-5p	0.60	0.11	hsa-miR-205-5p	0.82	0.07	hsa-miR-205-5p	0.46	0.35	hsa-miR-205-5p	0.30	0.68
hsa-miR-20a-5p	0.21	0.61	hsa-miR-20a-5p	0.57	0.13	hsa-miR-20a-5p	-0.03	0.99	hsa-miR-20a-5p	0.51	0.33
hsa-miR-20b-5p	0.11	0.80	hsa-miR-20b-5p	0.31	0.46	hsa-miR-20b-5p	-0.01	0.99	hsa-miR-20b-5p	0.23	0.68
hsa-miR-210-3p	0.42	0.83	hsa-miR-210-3p	1.10	0.58	hsa-miR-210-3p	-0.02	0.99	hsa-miR-210-3p	1.06	0.66
hsa-miR-21-5p	-0.06	0.83	hsa-miR-21-5p	-0.17	0.63	hsa-miR-21-5p	0.00	0.99	hsa-miR-21-5p	-0.22	0.66
hsa-miR-222-3p	0.29	0.40	hsa-miR-222-3p	0.44	0.21	hsa-miR-222-3p	0.20	0.68	hsa-miR-222-3p	0.20	0.66
hsa-miR-223-3p	-0.62	0.11	hsa-miR-223-3p	-0.59	0.21	hsa-miR-223-3p	-0.65	0.17	hsa-miR-223-3p	-0.01	0.97
hsa-miR-22-3p	0.43	0.18	hsa-miR-22-3p	0.95	0.01	hsa-miR-22-3p	0.11	0.93	hsa-miR-22-3p	0.84	0.01
hsa-miR-25-3p	1.01	0.00	hsa-miR-25-3p	1.01	0.00	hsa-miR-25-3p	1.00	0.00	hsa-miR-25-3p	-0.07	0.85
hsa-miR-26a-5p	-0.68	0.16	hsa-miR-26a-5p	-0.49	0.49	hsa-miR-26a-5p	-0.80	0.14	hsa-miR-26a-5p	0.27	0.71
hsa-miR-26b-5p	-0.20	0.75	hsa-miR-26b-5p	-0.29	0.62	hsa-miR-26b-5p	-0.15	0.93	hsa-miR-26b-5p	-0.21	0.69
hsa-miR-27a-3p	-0.35	0.25	hsa-miR-27a-3p	-0.21	0.58	hsa-miR-27a-3p	-0.43	0.25	hsa-miR-27a-3p	0.18	0.68
hsa-miR-27b-3p	-0.09	0.83	hsa-miR-27b-3p	-0.41	0.26	hsa-miR-27b-3p	0.10	0.99	hsa-miR-27b-3p	-0.60	0.66
hsa-miR-29a-3p	0.13	0.67	hsa-miR-29a-3p	0.38	0.15	hsa-miR-29a-3p	-0.03	0.99	hsa-miR-29a-3p	0.40	0.24
hsa-miR-29b-3p	0.46	0.11	hsa-miR-29b-3p	0.80	0.01	hsa-miR-29b-3p	0.24	0.57	hsa-miR-29b-3p	0.48	0.24
hsa-miR-29c-3p	0.44	0.10	hsa-miR-29c-3p	0.73	0.01	hsa-miR-29c-3p	0.25	0.48	hsa-miR-29c-3p	0.44	0.23
hsa-miR-328-3p	2.94	0.00	hsa-miR-328-3p	0.66	0.21	hsa-miR-328-3p	4.39	0.00	hsa-miR-328-3p	-3.92	0.00
hsa-miR-340-5p	0.60	0.67	hsa-miR-340-5p	0.07	0.95	hsa-miR-340-5p	0.93	0.65	hsa-miR-340-5p	-0.98	0.71
hsa-miR-424-5p	1.10	0.04	hsa-miR-424-5p	0.71	0.02	hsa-miR-424-5p	1.34	0.15	hsa-miR-424-5p	-0.68	0.66
hsa-miR-489-3p	1.06	0.00	hsa-miR-489-3p	0.88	0.01	hsa-miR-489-3p	1.17	0.00	hsa-miR-489-3p	-0.34	0.66
hsa-miR-497-5p	0.46	0.83	hsa-miR-497-5p	-1.83	0.33	hsa-miR-497-5p	1.91	0.62	hsa-miR-497-5p	-3.99	0.23
hsa-miR-7-5p	1.43	0.01	hsa-miR-7-5p	1.20	0.00	hsa-miR-7-5p	1.58	0.12	hsa-miR-7-5p	-0.53	0.69
hsa-miR-93-5p	0.50	0.11	hsa-miR-93-5p	0.79	0.02	hsa-miR-93-5p	0.32	0.45	hsa-miR-93-5p	0.39	0.55

Table S 1. List of 54 circulating miRNAs in the plasma, their differential expression in healthy control and breast cancer patients, with the appropriate log2 fold change (FC) and Adjusted p-value (BH) was listed in the table. The relative fold difference between any two groups was assessed by computing the (2(−ΔΔCt)) method as proposed by Livak et al.,2001. Data was analyzed by unpaired t-test. The two-tailed test p-values were corrected for multiple testing using Benjamini-Hochberg (BH) method to reduce the false discovery rate (FDR).

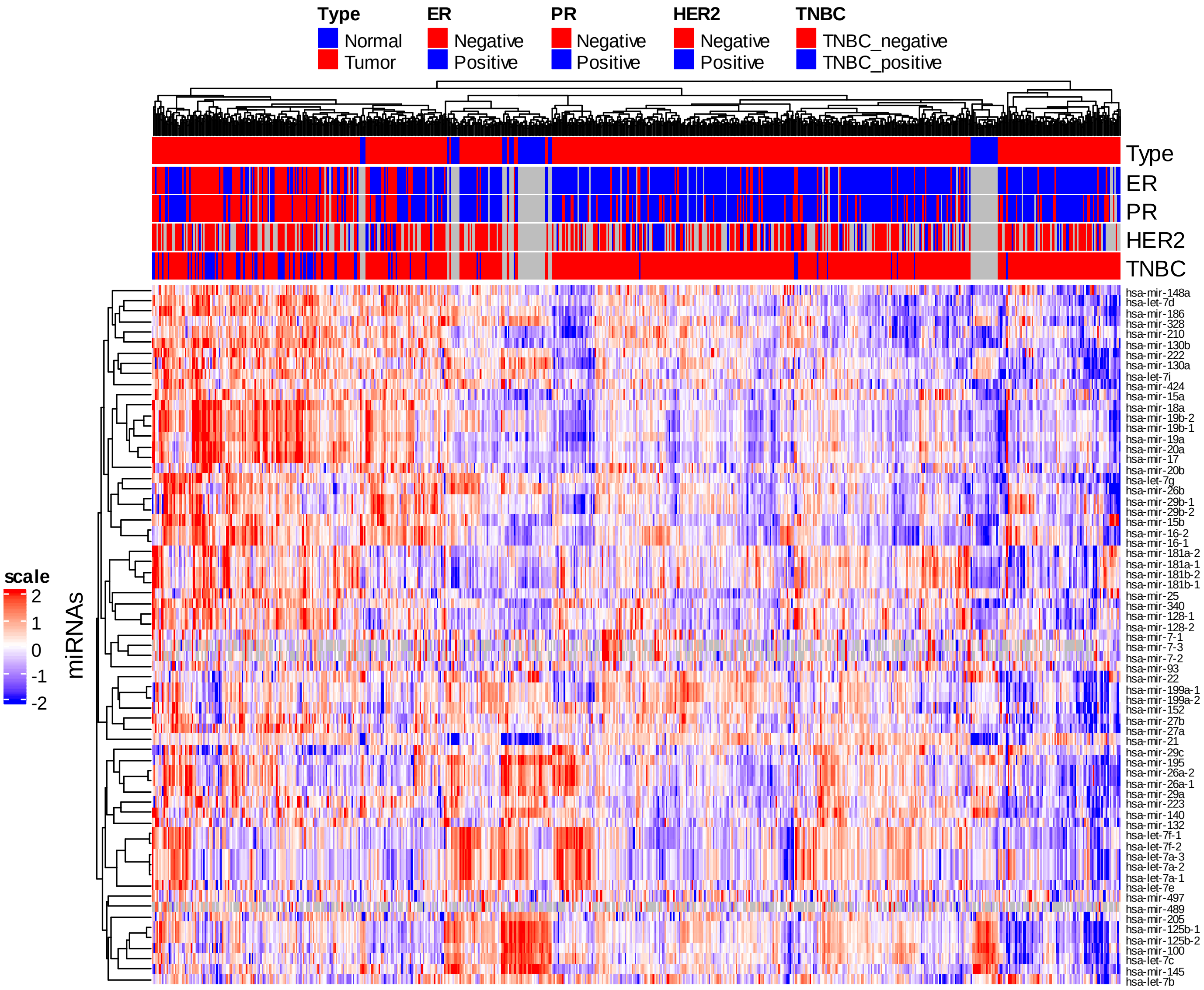

Figure S 1. Expression pattern of miRNAs in the TCGA-BRCA cohort. The heatmap shows the euclidean distance-based clustering and expression pattern of miRNAs across TCGA-BRCA cohort which includes 1180 solid tissue samples of breast cancer (n=1076) and disease-free individuals (n=104). The miRNA clustering tree is presented on the left, with the sample-clustering tree at the top. Clinical information related to ER, PR, HER2 and TNBC status of the patients are provided above the heatmap. The color scale presented illustrates the relative expression level of the miRNAs: red, high expression; blue, low expression.

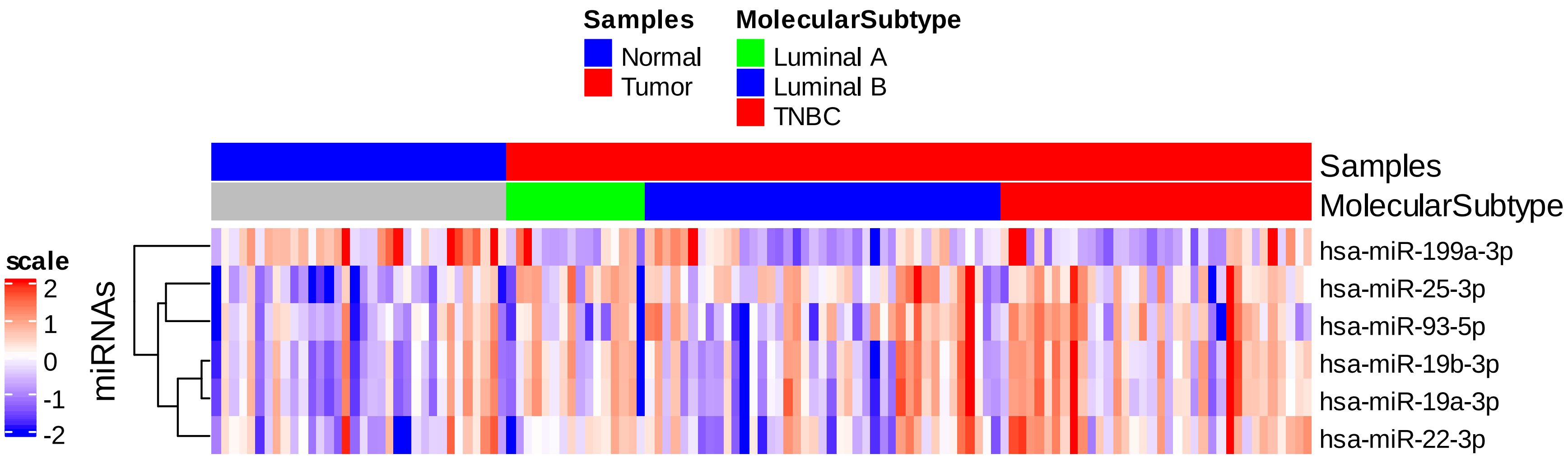

Figure S 2. Hierarchical clustering and heatmap of miRNAs across samples shows the expression pattern of significant differentially expressed circulating miRNAs across normal and different subtypes of breast cancers. Red and blue colors in the heatmap indicate, high and low expression of miRNAs respectively. The colored bars above the heatmap provide sample information. The miRNA clustering tree is presented on the left, with the samples at the top. The color scale presented illustrates the relative expression level of the miRNAs: red, high expression; blue, low expression.

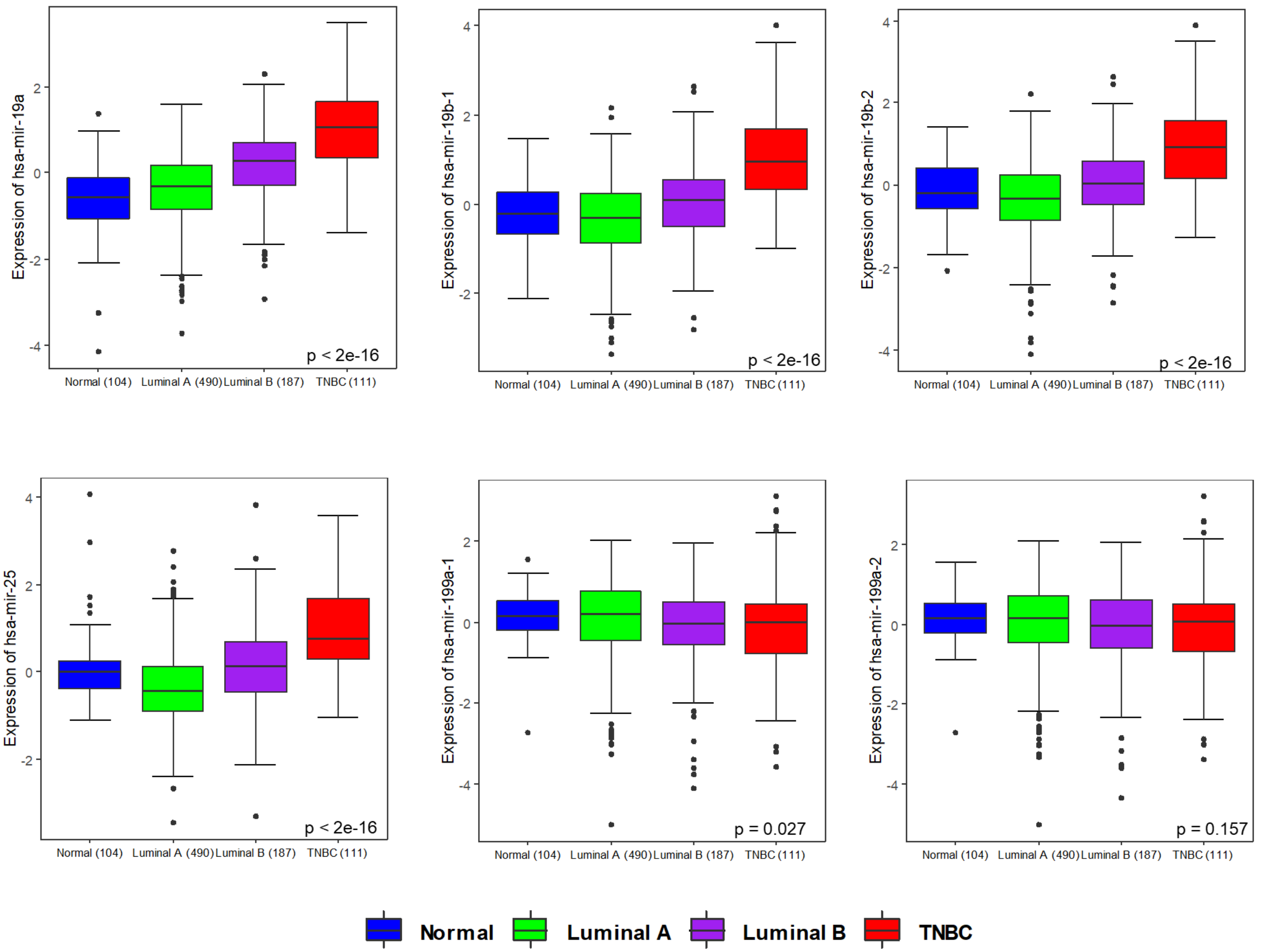

Figure S 3. Whisker plots of comparisons between miRNA expression levels in different subtypes of breast cancer and normal healthy profiled from TCGA.

Group Classifications	miRNA ID	Average Normal expression ± SD	Average Tumor expression ± SD	Log2 FC Cancer vs Normal	Regulation Up/Down	p-value (unpaired t-test)	Adjusted p-value (BH)
**All BC vs. Control**	hsa-let-7b-5p	28.033 ± 1.192	26.875 ± 1.185	1.157	UP	9.5×10^{-6}	1.2×10^{-4}
	hsa-miR-25-3p	26.765 ± 0.928	25.757 ± 0.928	1.008	UP	1.2×10^{-6}	2.1×10^{-5}
	hsa-miR-16-5p	22.816 ± 0.907	21.917 ± 1.031	0.9	UP	1.1×10^{-5}	1.2×10^{-4}
	hsa-let-7i-5p	27.667 ± 1.028	26.952 ± 0.915	0.716	UP	7.6×10^{-4}	4.5×10^{-3}
	hsa-miR-199a-3p	28.493 ± 1.548	29.982 ± 1.646	-1.49	Down	1.4×10^{-5}	1.2×10^{-4}
**TNBC vs. Control**	hsa-miR-25-3p	26.765 ± 0.928	25.752 ± 0.965	1.013	UP	3.0×10^{-5}	8.1×10^{-4}
	hsa-miR-22-3p	26.433 ± 1.273	25.487 ± 0.976	0.946	UP	9.4×10^{-4}	5.7×10^{-3}
	hsa-miR-19b-3p	26.138 ± 0.998	25.239 ± 0.953	0.898	UP	2.7×10^{-4}	2.7×10^{-3}
	hsa-miR-19a-3p	25.447 ± 1.003	24.590 ± 0.960	0.857	UP	5.1×10^{-4}	3.5×10^{-3}
	hsa-miR-93-5p	27.430 ± 1.094	26.639 ± 1.236	0.791	UP	6.0×10^{-3}	2.2×10^{-2}
	hsa-miR-199a-3p	28.493 ± 1.548	29.985 ± 1.724	-1.493	Down	3.0×10^{-4}	2.7×10^{-3}
	hsa-miR-210-3p	32.757 ± 6.075	31.653 ± 5.702	1.103	UP	4.4×10^{-1}	5.8×10^{-1}
	hsa-miR-16-5p	22.816 ± 0.907	21.676 ± 0.888	1.14	UP	1.32×10^{-6}	7.1×10^{-5}

Table S 2. Circulating miRNAs in the plasma that were differentially expressed in healthy control and breast cancer patients, with fold change (FC), p-value (unpaired t-test), Adjusted p-value (BH) < 0.05. Total miRNA was isolated from plasma samples and the mature miRNAs was determined by the miScript PCR System. The relative fold difference between any two groups was assessed by computing ($2(-\Delta\Delta Ct)$) method as proposed by Livak et al.,2001. Data was analyzed by unpaired t-test. The two-tailed test p-values were corrected for multiple testing using Benjamini-Hochberg (BH) method to reduce the false discovery rate (FDR).

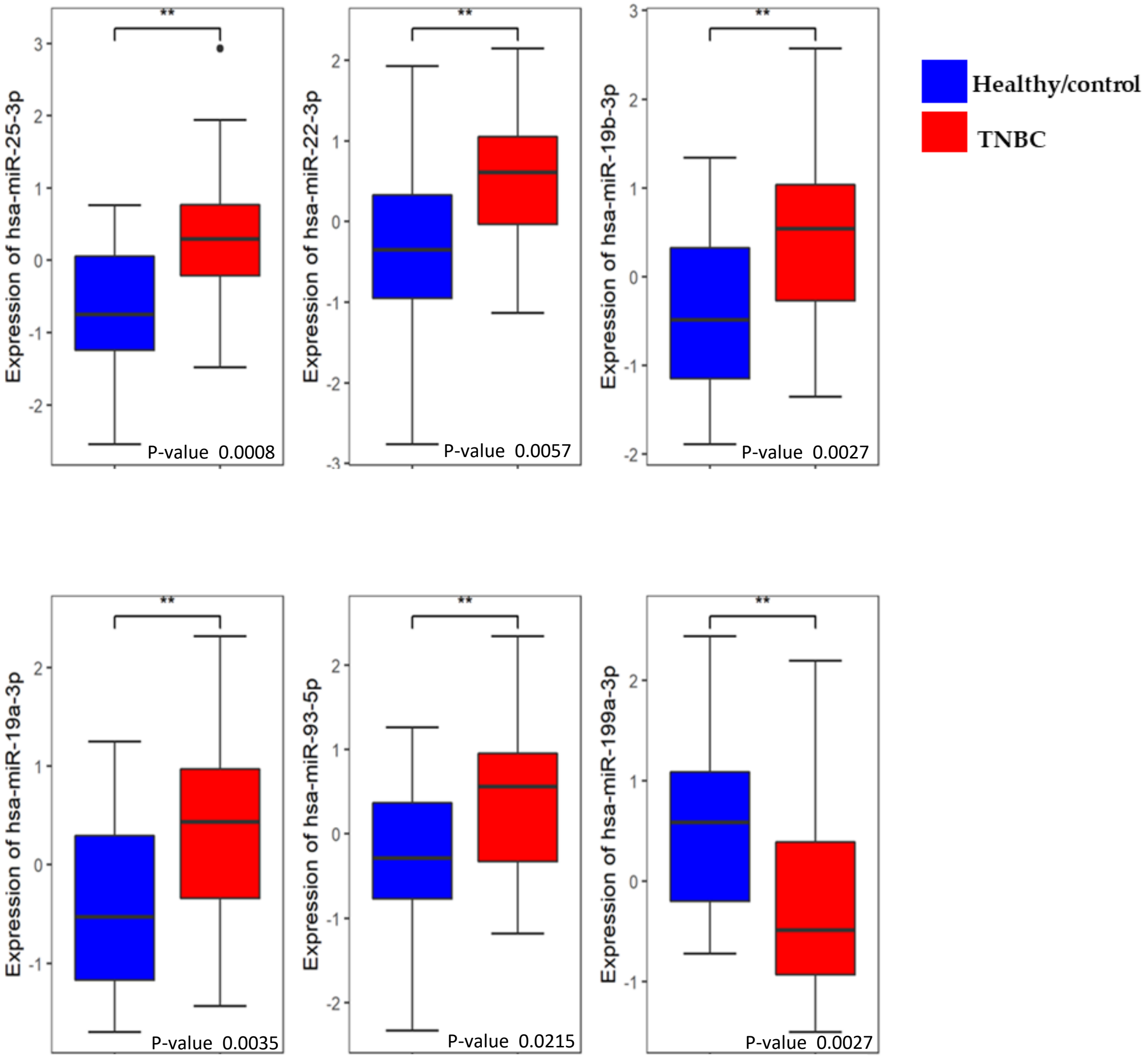

Figure S 4. Whisker plots of comparisons between miRNA expression levels in normal control and triple negative breast cancer patients samples (TNBC). a miR-19a/b-3p, miR-25-3p, miR-22-3p, miR-93-5p, and miR-210-3p were up regulated compared to that found in normal and miR-199a-3p as a significantly downregulated plasma miRNA . The relative fold difference between any two groups was calculated by (2(−ΔΔCt)) method as proposed by Livak et al.,2001. Data was analyzed by unpaired t-test. The two-tailed test p-values were corrected for multiple testing using Benjamini-Hochberg (BH) method to reduce the false discovery rate (FDR).

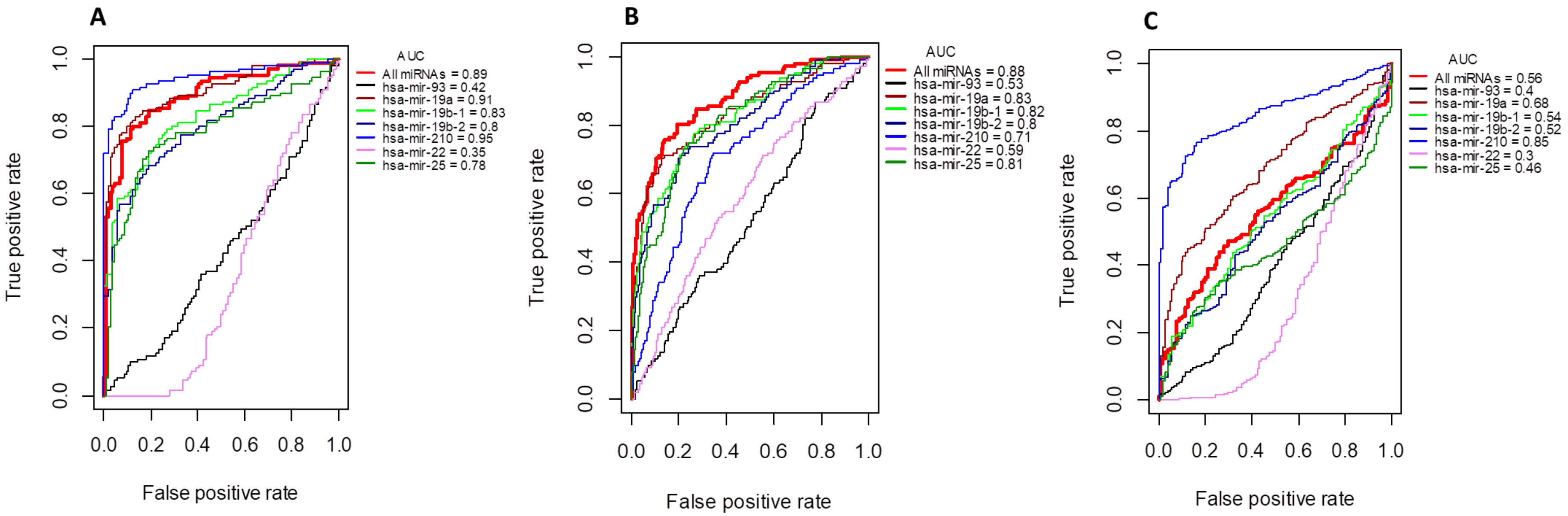

Figure S 5. Receiver Operating Characteristic (ROC) curves show the discriminatory ability of significant differentially regulated miRNAs across (A) TNBC vs Control, (B) TNBC vs luminal samples. (C) All breast cancer tumors vs. Normal samples. ROC curves of the individual miRNAs are shown in different colors. AUC values corresponding to the individual miRNAs are provided in the plot.

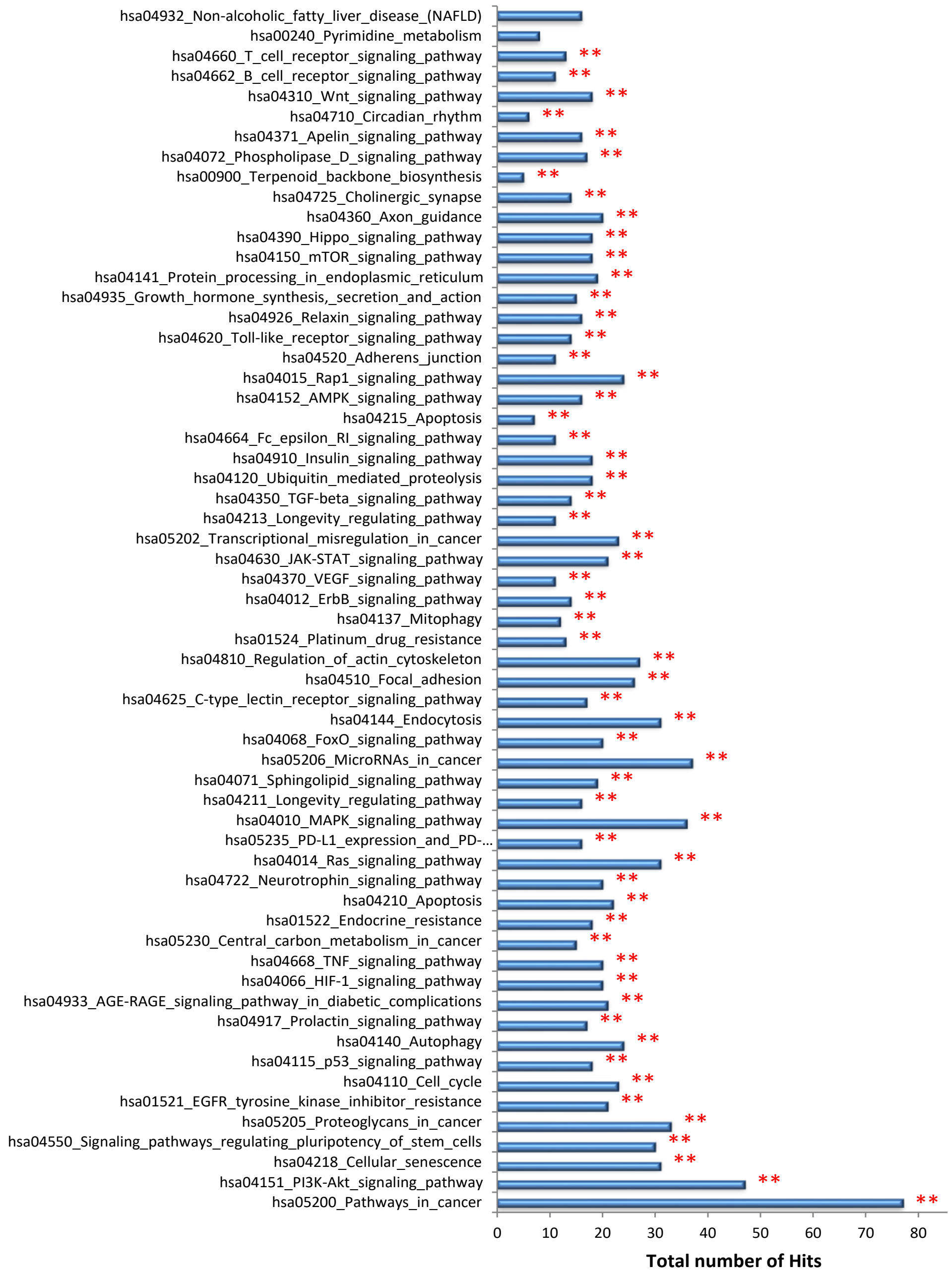

Figure S 6. Enriched KEGG signaling pathways in TNBC at the 3'-UTR of target genes with adjusted p-values (BH) ≤0.05. Standard enrichment analysis based on the hypergeometric tests (fisher-exact-test),p-values shown in the figure are as follows:** p-value (BH) ≤0.05.

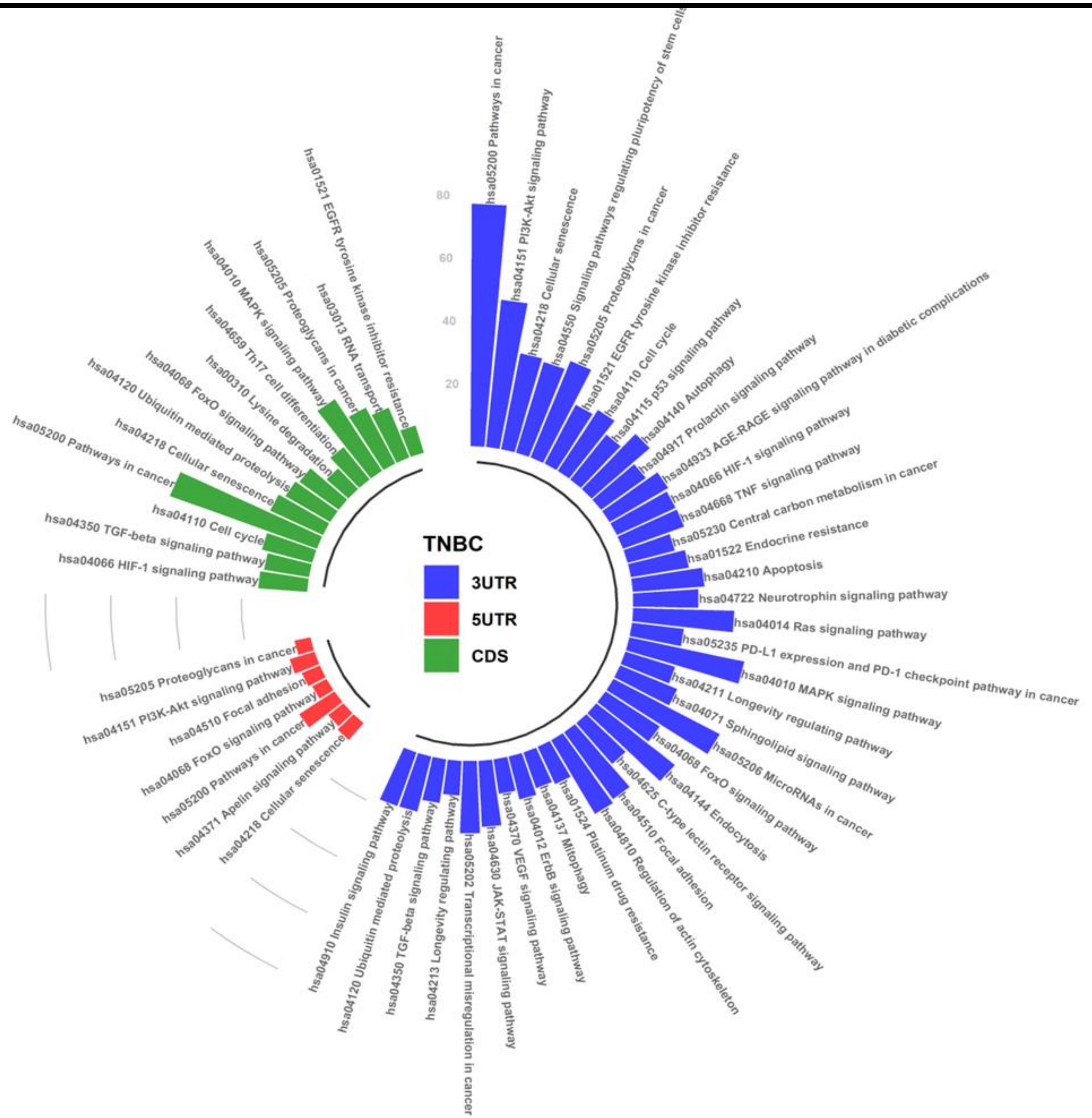

Figure S 7. Significantly enriched Kyoto Encyclopedia of Genes and Genomes (KEGG) signaling pathways with the adjusted p-value ≤ 0.05, among triple negative breast cancer (TNBC) arranged by 3'-UTR, 5'-UTR and CDS region targeting are shown as a circular bar-chart. Standard enrichment analysis based on the hypergeometric tests (fisher-exact-test) was performed, the enriched KEGG pathways based on p-value were filtered and shown as circular bar chart.

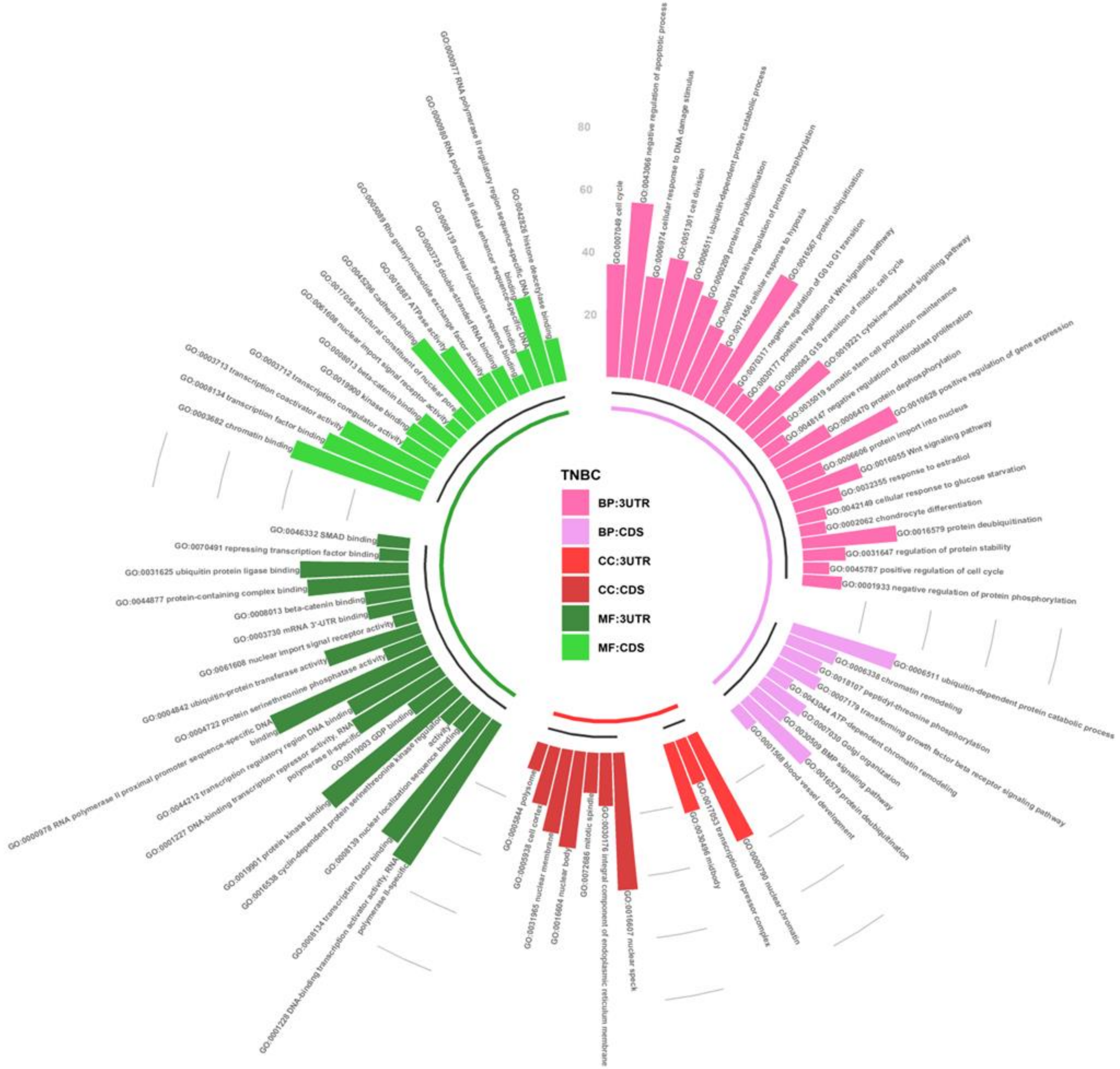

Figure S 8. Gene ontologies enriched in TNBC is shown as circular bar chart. Significant Biological Process (BP), Cellular Component (CC) and Molecular Functions (MF) with the adjusted p-value ≤ 0.05, enriched in 3'-UTR, 5'-UTR and CDS specific entries for triple negative breast cancer (TNBC) are selected from the standard enrichment analysis based on the hypergeometric tests (fisher-exact-test), and visualized as circular bar chart.

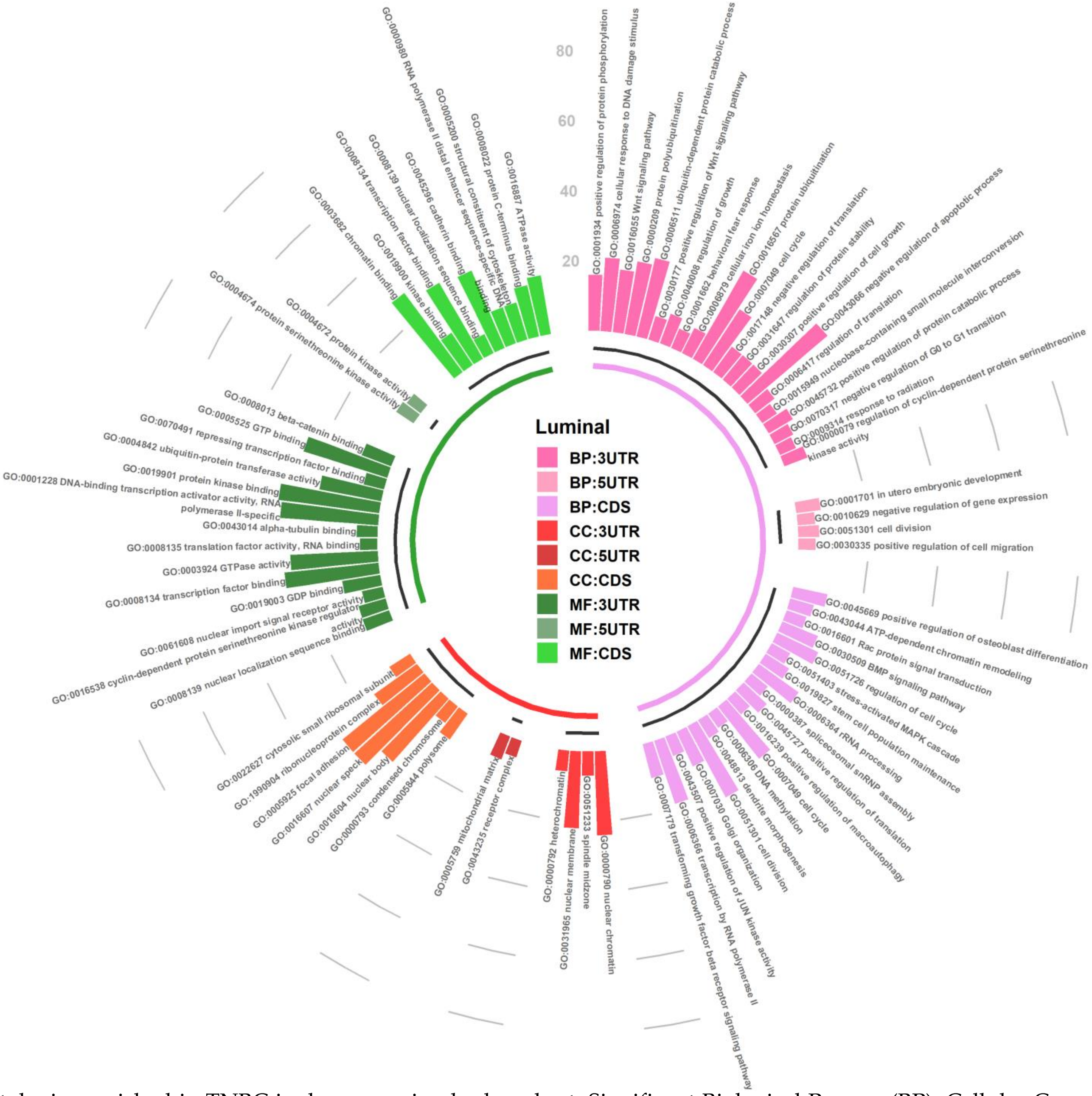

Figure S 9. Gene ontologies enriched in TNBC is shown as circular bar chart. Significant Biological Process (BP), Cellular Component (CC) and Molecular Functions (MF) with the adjusted p-value ≤ 0.05, enriched in 3UTR and CDS specific entries for luminal breast cancer are selected from the standard enrichment analysis based on the hypergeometric tests (fisher-exact-test), and visualized as circular bar chart.

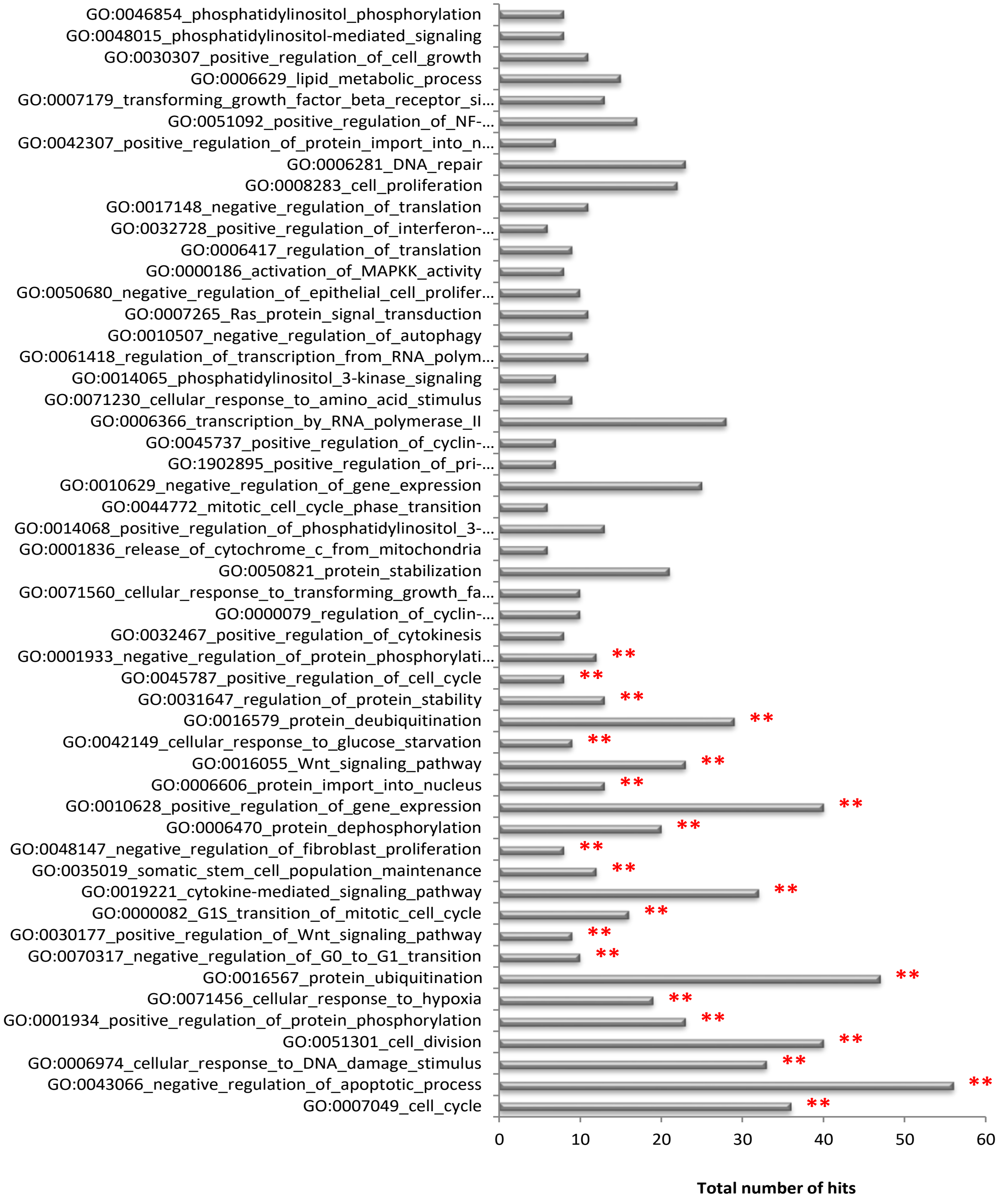

Figure S 10. Gene ontologies enriched in triple negative breast cancer (TNBC) . Significant Biological Process (BP) entries for TNBC with adjusted p-value (BH) ≤ 0.05. Standard enrichment analysis based on the hypergeometric tests (fisher-exact-test). p-values shown in the figure are as follows:** p-value (BH) ≤0.05.

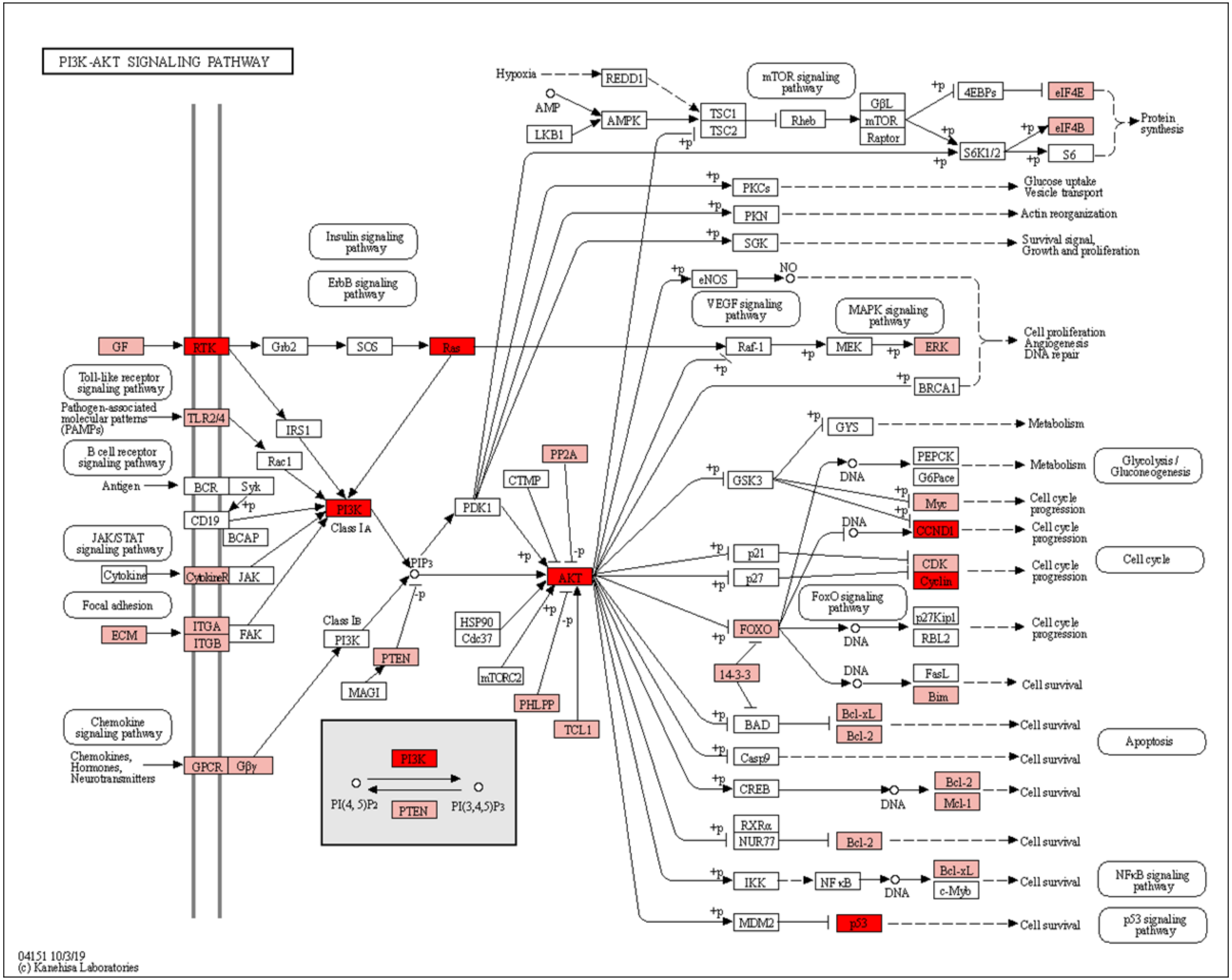

Figure S 11. PI3K-AKT signaling KEGG pathway with target genes of significant differentially expressed miRNAs in triple negative breast cancer (TNBC) highlighted . Red color represents proteins encoded by genes known to be targets of significant miRNAs. A darker color indicates a greater number of miRNAs that target the gene encoding that specific protein. If the protein is white, no miRNAs target the encoding gene.

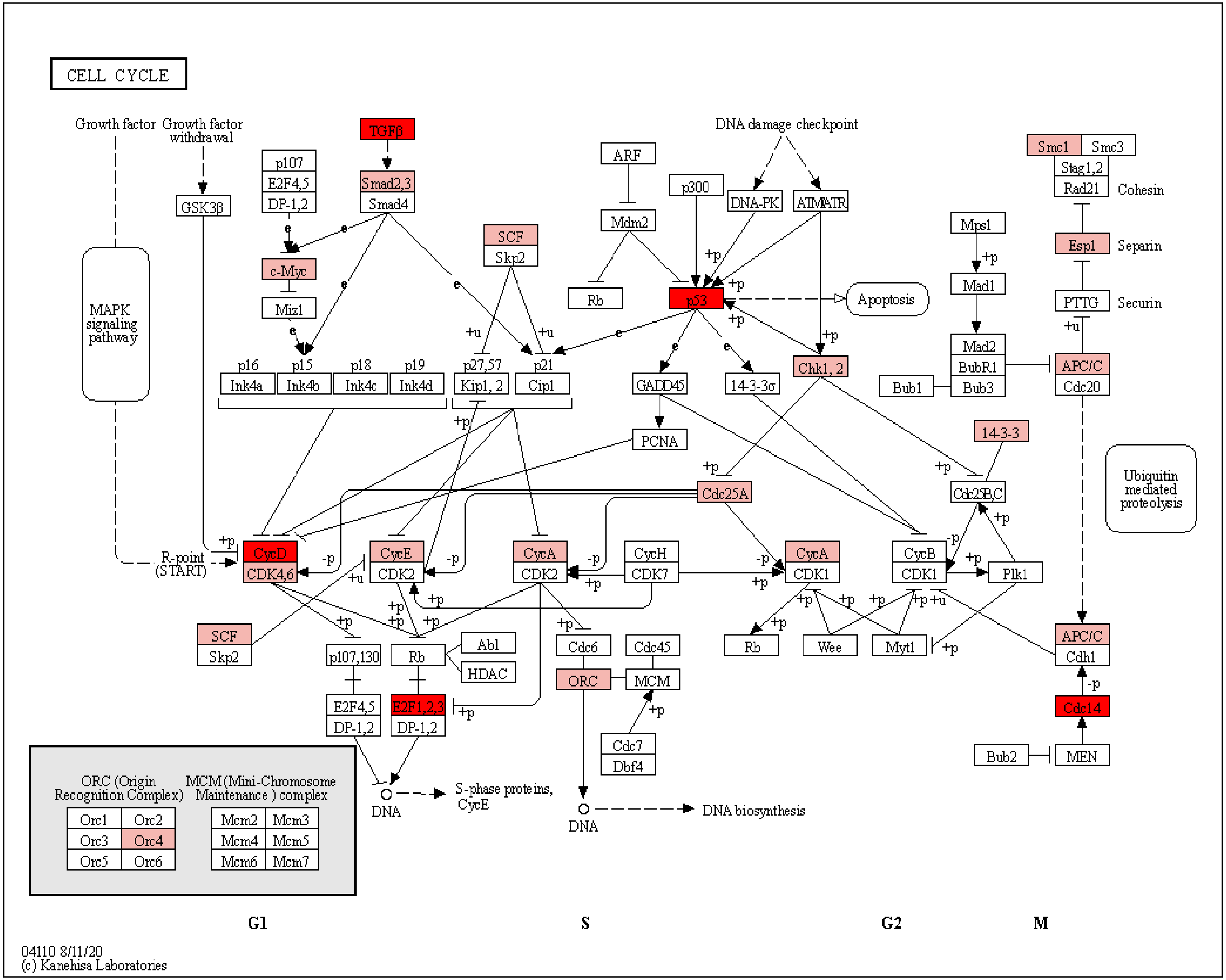

Figure S 12. Cell cycle KEGG with target genes of significant differentially expressed miRNAs in triple negative breast cancer (TNBC) highlighted . Red color represents proteins encoded by genes known to be targets of significant miRNAs. A darker color indicates a greater number of miRNAs that target the gene encoding that specific protein. If the protein is white, no miRNAs target the encoding gene.

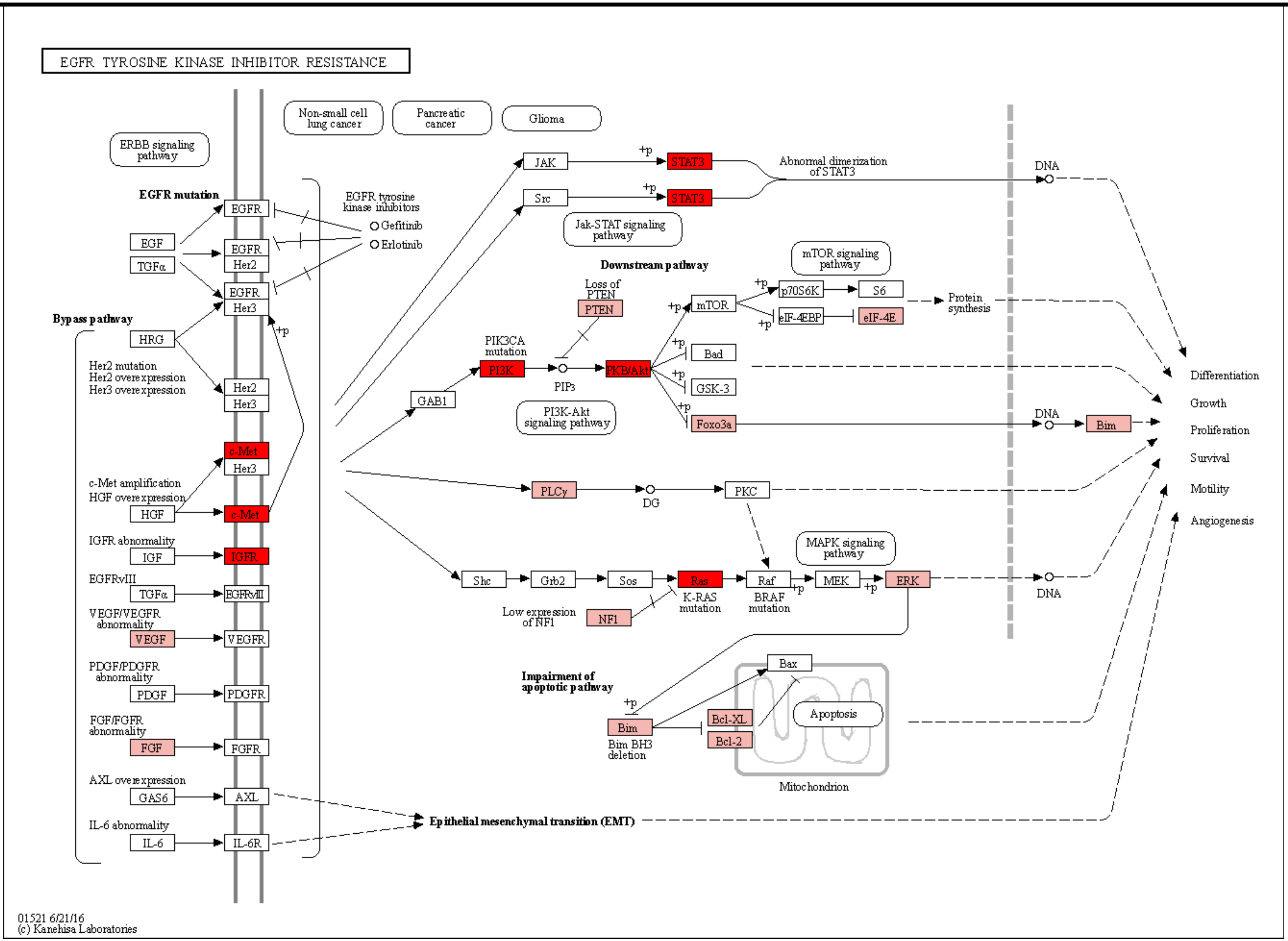

Figure S 13. EGFR tyrosine kinase inhibitor resistance signaling (TKIs) KEGG pathway with target genes of significant differentially expressed miRNAs in triple negative breast cancer (TNBC) highlighted . Red color represents proteins encoded by genes known to be targets of significant miRNAs. A darker color indicates a greater number of miRNAs that target the gene encoding that specific protein. If the protein is white, no miRNAs target the encoding gene.

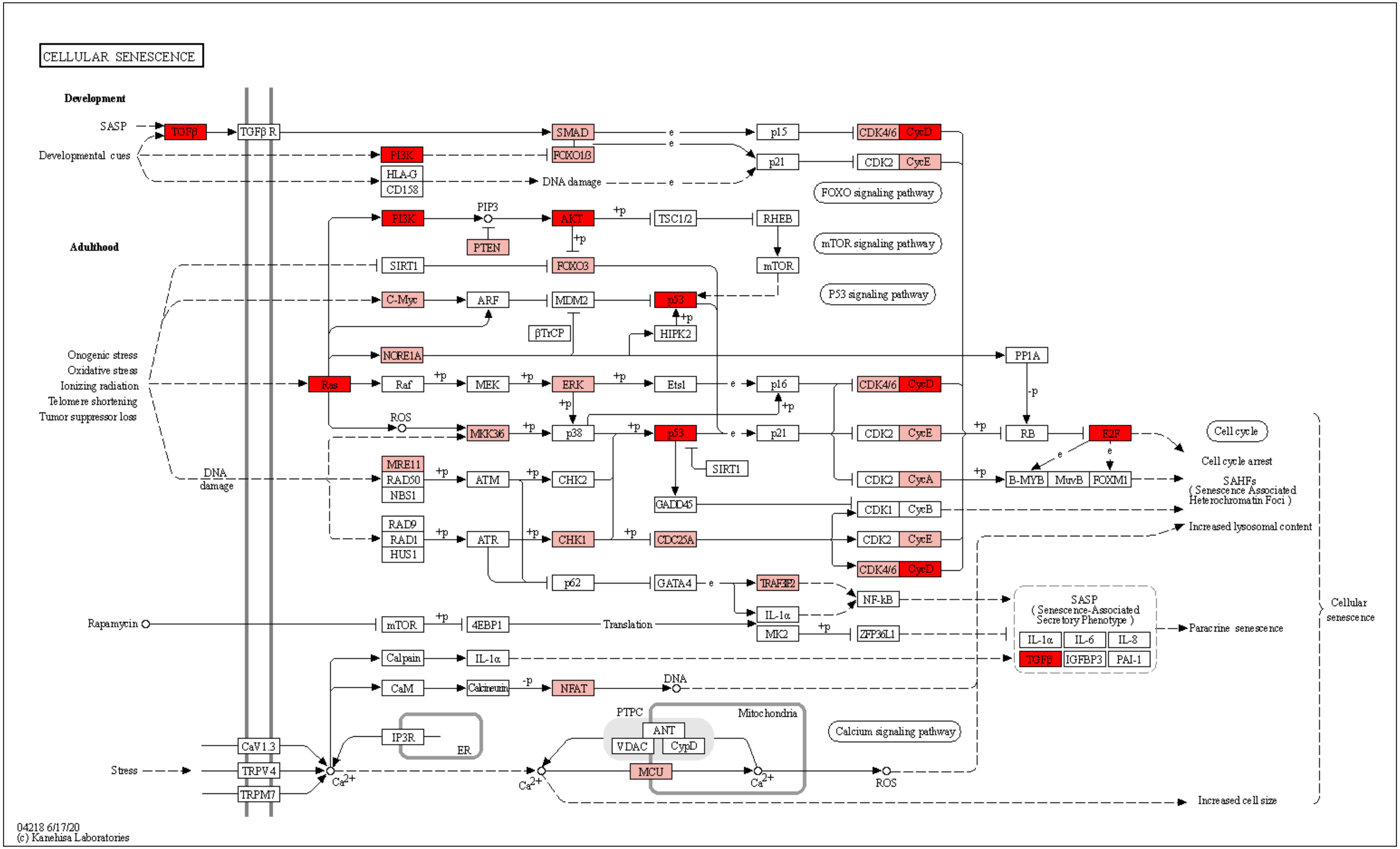

Figure S 14. Cellular senescence signaling KEGG pathway with target genes of significant differentially expressed miRNAs in triple negative breast cancer (TNBC) highlighted . Red color represents proteins encoded by genes known to be targets of significant miRNAs. A darker color indicates a greater number of miRNAs that target the gene encoding that specific protein. If the protein is white, no miRNAs target the encoding gene.

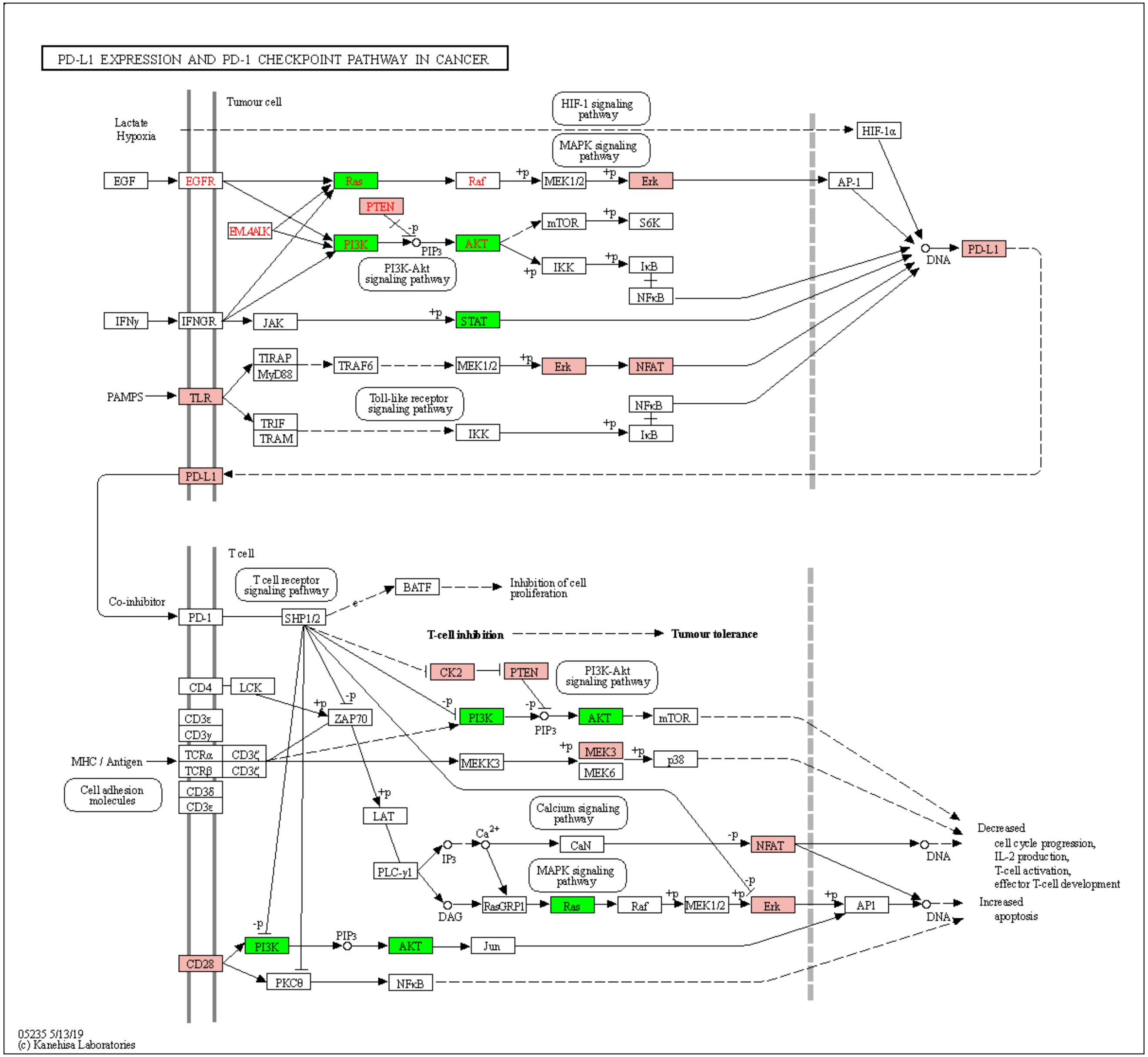

Figure S 15. PD-L1 and PD-1 checkpoint pathway KEGG with target genes of significant differentially expressed miRNAs in triple negative breast cancer (TNBC) highlighted . Colors represent proteins encoded by genes known to be targets of significant miRNAs. The green color indicates a greater number of miRNAs that target the gene encoding that specific protein. If the protein is white, no miRNAs target the encoding gene.

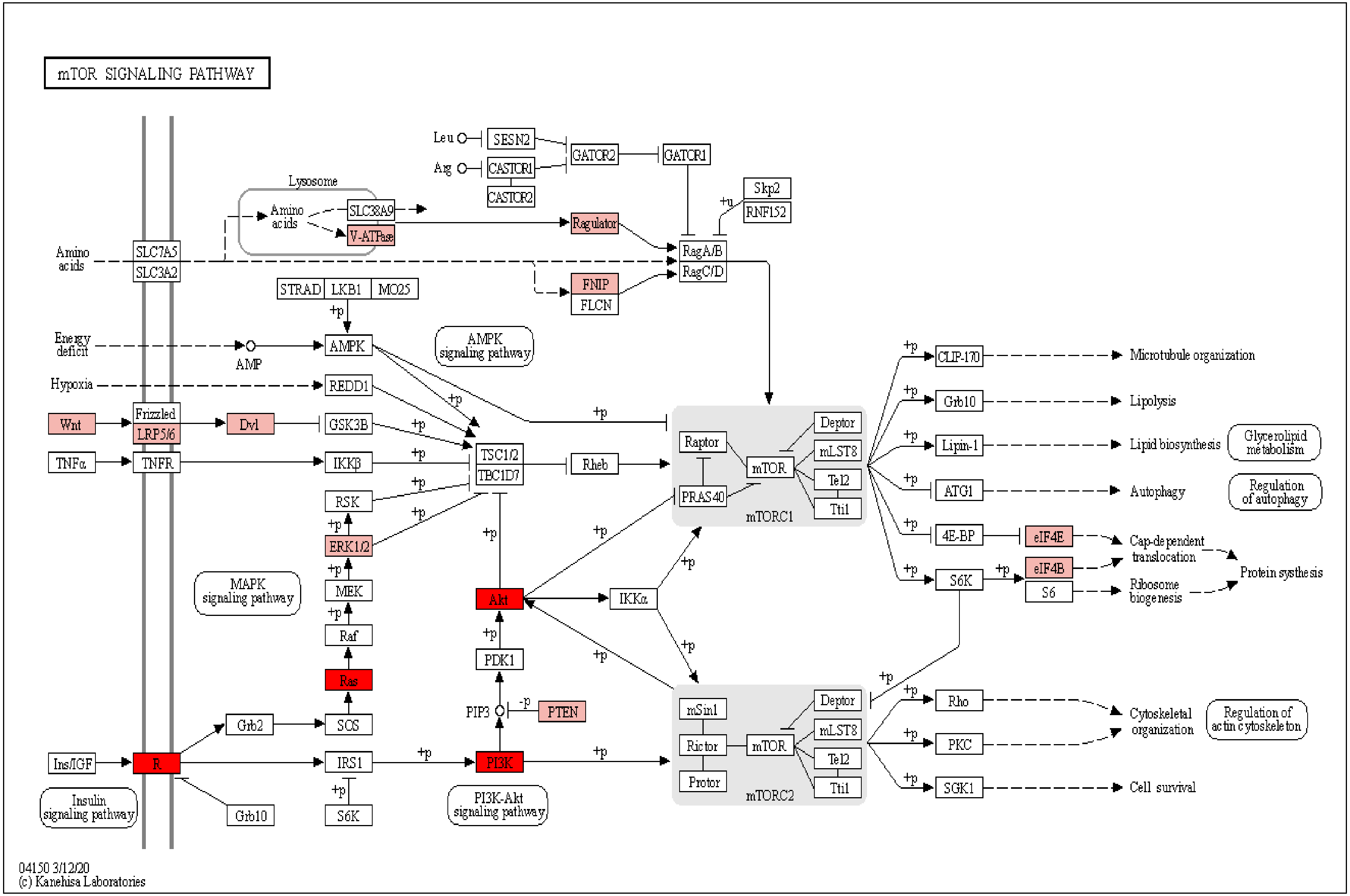

Figure S 16. mTOR signaling KEGG pathway with target genes of significant differentially expressed miRNAs in triple negative breast cancer (TNBC) highlighted . Red color represents proteins encoded by genes known to be targets of significant miRNAs. A darker color indicates a greater number of miRNAs that target the gene encoding that specific protein. If the protein is white, no miRNAs target the encoding gene.

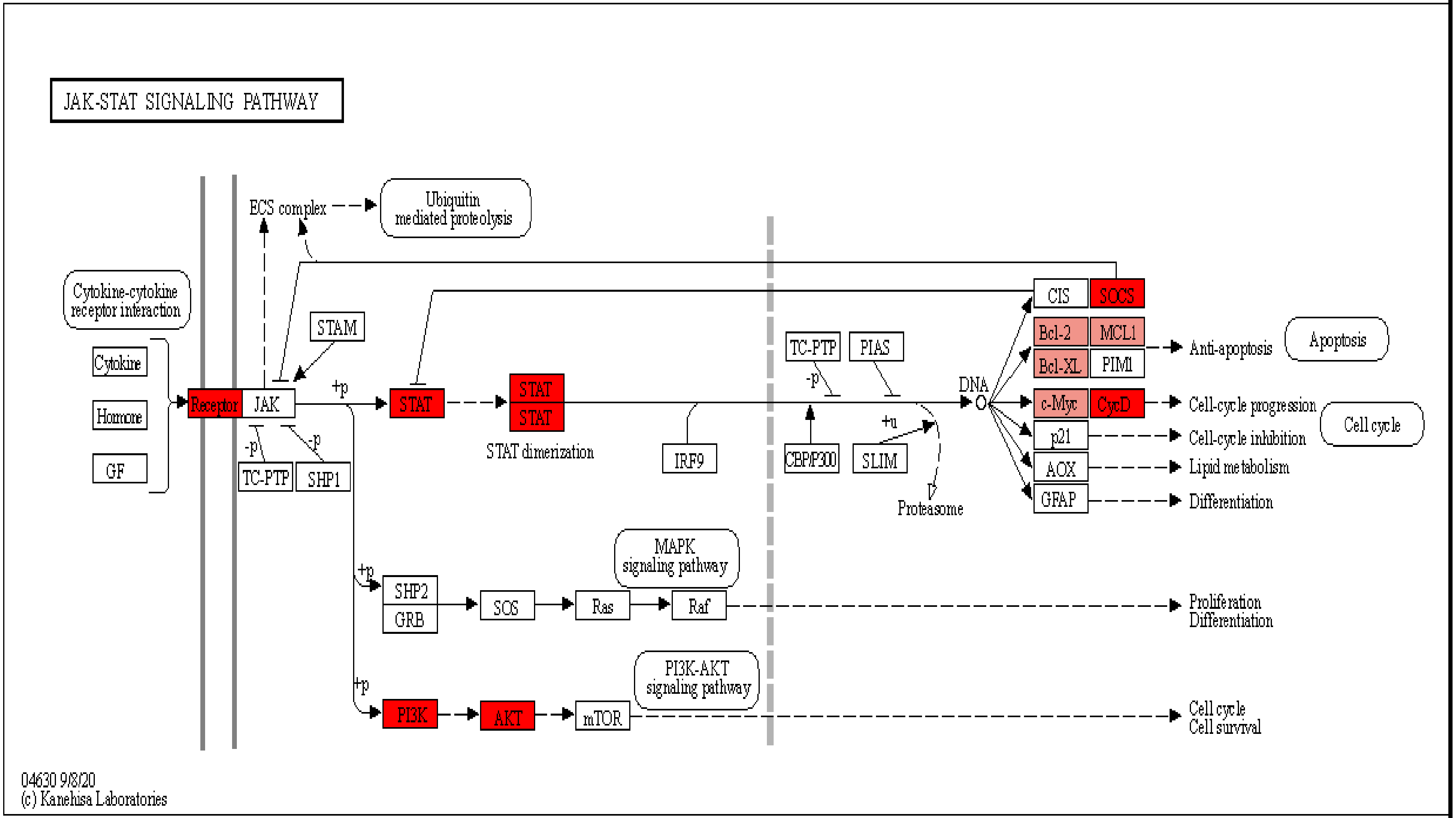

Figure S 17. JAK/STAT signaling KEGG pathway with target genes of significant differentially expressed miRNAs in triple negative breast cancer (TNBC) highlighted . Red color represents proteins encoded by genes known to be targets of significant miRNAs. A darker color indicates a greater number of miRNAs that target the gene encoding that specific protein. If the protein is white, no miRNAs target the encoding gene.

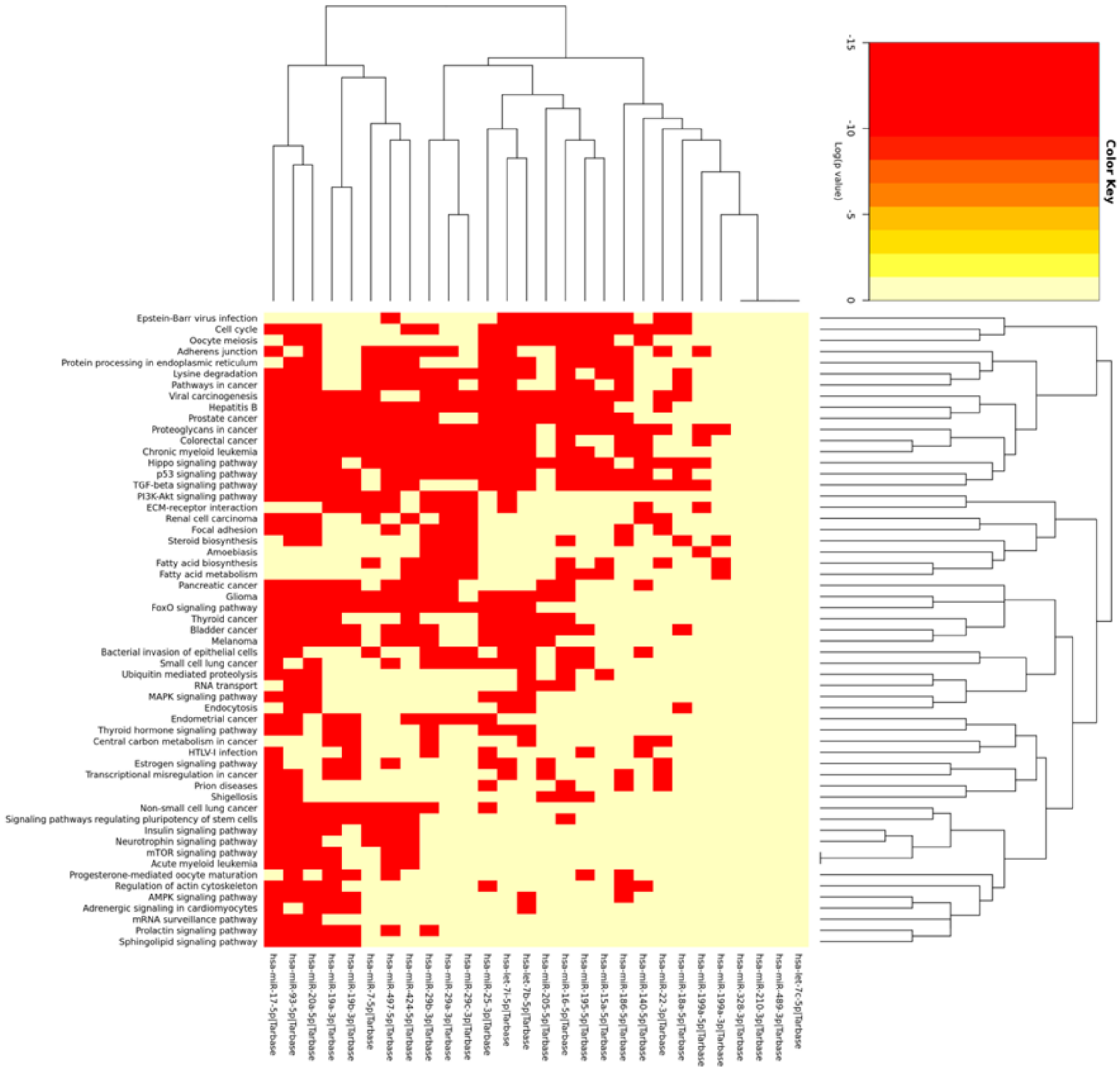

Figure S 18. Hierarchical Clusters of miRNAs/KEGG targeted pathways regulated by significant differentially regulated miRs in TNBC based on the existence or absence of statistical significance using Fisher exact test (Hypergeometric distribution) and a p-value ≤0.05.
